# Interpretability Versus Accuracy: A Comparison of Machine Learning Models Built Using Different Algorithms, Performance Measures, and Features to Predict *E. coli* Levels in Agricultural Water

**DOI:** 10.3389/frai.2021.628441

**Published:** 2021-05-14

**Authors:** Daniel L. Weller, Tanzy M. T. Love, Martin Wiedmann

**Affiliations:** ^1^ Department of Biostatistics and Computational Biology, University of Rochester, Rochester, NY, United States; ^2^ Department of Food Science, Cornell University, Ithaca, NY, United States; ^3^ Current Affiliation, Department of Environmental and Forest Biology, SUNY College of Environmental Science and Forestry, Syracuse, NY, United States

**Keywords:** *E. coli*, machine learning, predictive model, food safety, water quality

## Abstract

Since *E. coli* is considered a fecal indicator in surface water, government water quality standards and industry guidance often rely on *E. coli* monitoring to identify when there is an increased risk of pathogen contamination of water used for produce production (e.g., for irrigation). However, studies have indicated that *E. coli* testing can present an economic burden to growers and that time lags between sampling and obtaining results may reduce the utility of these data. Models that predict *E. coli* levels in agricultural water may provide a mechanism for overcoming these obstacles. Thus, this proof-of-concept study uses previously published datasets to train, test, and compare *E. coli* predictive models using multiple algorithms and performance measures. Since the collection of different feature data carries specific costs for growers, predictive performance was compared for models built using different feature types [geospatial, water quality, stream traits, and/or weather features]. Model performance was assessed against baseline regression models. Model performance varied considerably with root-mean-squared errors and Kendall’s Tau ranging between 0.37 and 1.03, and 0.07 and 0.55, respectively. Overall, models that included turbidity, rain, and temperature outperformed all other models regardless of the algorithm used. Turbidity and weather factors were also found to drive model accuracy even when other feature types were included in the model. These findings confirm previous conclusions that machine learning models may be useful for predicting when, where, and at what level *E. coli* (and associated hazards) are likely to be present in preharvest agricultural water sources. This study also identifies specific algorithm-predictor combinations that should be the foci of future efforts to develop deployable models (i.e., models that can be used to guide on-farm decision-making and risk mitigation). When deploying *E. coli* predictive models in the field, it is important to note that past research indicates an inconsistent relationship between *E. coli* levels and foodborne pathogen presence. Thus, models that predict *E. coli* levels in agricultural water may be useful for assessing fecal contamination status and ensuring compliance with regulations but should not be used to assess the risk that specific pathogens of concern (e.g., *Salmonella*, *Listeria*) are present.

## Introduction

Following a 2018 Shiga-toxin producing *Escherichia coli* outbreak linked to romaine lettuce, investigators identified irrigation water contaminated by cattle feces as the probable source (Bottichio et al., 2019). Such a conclusion is not uncommon, and fecal contamination of surface water has been repeatedly identified as the probable cause of enteric disease outbreaks (Johnson, 2006; Ackers et al., 1998; Wachtel et al., 2002; Greene et al., 2008; Barton Behravesh et al., 2011; Food and Drug Administration, 2019; Food and Drug Administration, 2020). As a result, non-pathogenic fecal indicator bacteria (FIBs), like *E. coli*, are used to assess when and where fecal contaminants, including food and waterborne pathogens, may be present in agricultural and recreational waterways. Indeed, many countries and industry groups have established standards for agricultural and/or recreational surface water based on FIB levels; when samples are above a binary cut-off the probability of fecal contamination is deemed sufficient to require corrective action (Health Canada, 2012; US FDA, 2015; Health Canada, 2012; California Leafy Greens Marketing Agreement, 2017; Environmental Protection Agency, 2012; Corona et al., 2010; UK EA; EU Parliament, 2006; SA DWAF, 1996) For instance, the Australian and New Zealand governments established trigger values for thermotolerant coliforms in water applied to food and non-food crops (ANZECC, 2000), while the United States Produce Safety Rule (PSR) proposed an *E. coli*-based standard for surface water sources used for produce production (US FDA, 2015). Similarly, the California Leafy Greens Marketing Agreement requires *E. coli* testing for determining the microbial quality of water used for produce production (California Leafy Greens Marketing Agreement, 2017). However, multiple studies have suggested that the frequency of sampling required by the PSR and similar regulations may not be sufficient to capture spatiotemporal variability in microbial water quality (Edge et al., 2012; McEgan et al., 2013; Havelaar et al., 2017; Weller et al., 2020c). Thus, supplementary or alternative approaches for monitoring surface water for potential public health hazards may be needed (Edge et al., 2012; McEgan et al., 2013; Havelaar et al., 2017; Weller et al., 2020c).

While an alternative to current monitoring practices is to more frequently measure FIB levels in the waterway (e.g., immediately before each irrigation event), studies that quantified costs associated with the United States PSR found that the low-frequency testing proposed by the PSR presented a substantial economic burden to growers (Calvin et al., 2017; Astill et al., 2018). Additional concerns about the feasibility of water testing (e.g., access/proximity to labs), and the time lag between sampling and time of water use (minimum of 24 h) have also been raised (Havelaar et al., 2017; Wall et al., 2019; Weller et al., 2020c). Indeed, a study that sampled recreational waterways in Ohio over consecutive days found that a predictive model was able to better predict *E. coli* levels than using *E. coli* levels from samples collected on the day preceding sample collection (i.e., 24 h before) as the prediction; (Brady et al., 2009). Predictive models may thus provide an alternative or supplementary approach to *E. coli*-based monitoring of agricultural and recreational surface water sources.

While past studies have shown that predictive models can be useful for assessing public health hazards in recreational water (Olyphant, 2005; Hou et al., 2006; Brady and Plona, 2009; Hamilton and Luffman, 2009; Francy et al., 2013; Francy et al., 2014; Dada and Hamilton, 2016; Dada, 2019; Rossi et al., 2020), no models, to the author’s knowledge, have been developed to predict *E. coli* levels in surface water used for produce production (e.g., for irrigation, pesticide application, dust abatement, frost protection). Moreover, many of the recreational water quality studies only considered one algorithm during model development (e.g., (Olyphant, 2005; Brady et al., 2009; Brady and Plona, 2009; Hamilton and Luffman, 2009), including algorithms [e.g., regression, (Olyphant, 2005; Brady et al., 2009; Brady and Plona, 2009; Hamilton and Luffman, 2009)], which has more assumptions and may be less accurate than alternate algorithms (e.g., ensemble methods, support vector machines, (Kuhn and Johnson, 2016; Weller et al., 2020a)). As such, there is limited data on 1) how models for predicting *E. coli* levels in agricultural water should be implemented and validated, or 2) how the data used to train these models should be collected (e.g., types of features to focus data collection efforts on). Addressing these knowledge gaps is key if the aim is to develop and deploy field-ready models (models that can be used to create a cost-effective tool with a GUI interface, incorporated into growers’ food safety plans, and used to guide on-farm decision-making in real-time). Thus, there is a specific need for studies that assess and compare the efficacy of models built using different algorithms and different features (e.g., weather, water quality). This latter point is particularly important since the collection of each feature type carries specific costs, including time and capital investment, worker training/expertize, and computational costs. For example, growers can often easily obtain, with no capital investment, weather data from publicly accessible stations (e.g., airport stations, AZMet [cals.arizona.edu/AZMET]), however, since these stations are unlikely to be located at a given farm, the utility of these data for training accurate predictive models need to be determined. Conversely, growers can collect physicochemical water quality data on-site provided they invest in equipment (e.g., water quality probes) and train staff to use the equipment. This proof-of-concept study aims to address these knowledge gaps and provide a framework on which future studies focused on developing field-ready models can build. Specifically, the objectives of this study were to 1) develop, assess, and compare the ability of models built using different algorithms and different combinations of feature types (e.g., geospatial, water quality, weather, and/or stream traits) to predict *E. coli* levels, and 2) highlight how model interpretation is affected by the performance measure used. Since this is a proof-of-concept and not an empirical, study that used previously published data, the focus of the current paper is on identifying and comparing different algorithms, performance measures, and feature sets, and not on developing a deployable model, or characterizing relationships between *E. coli* levels and features. The overarching aim of this paper is to provide a conceptual framework on which future studies can build, and to highlight what future studies should consider when selecting algorithms, performance measures, and feature sets.

It is also important to remember when interpreting the findings presented here, that past research indicates an inconsistent relationship between *E. coli* levels and foodborne pathogen presence (Harwood et al., 2005; McEgan et al., 2013; Pachepsky et al., 2015; Antaki et al., 2016; Weller et al., 2020c). Thus, *E. coli* models, like those developed here, may be useful for assessing fecal contamination status and ensure compliance with regulations but should not be used to determine if specific pathogens of concern (e.g., *Salmonella*, *Listeria*) are present. Since *E. coli* is used outside food safety as an indicator of fecal contamination (e.g., for recreational water), the findings from this study may have implications for mitigating other public health concerns as well.

## Materials and Methods

### Study Design and *E. coli* Enumeration

Existing datasets collected in 2018 (Weller et al., 2020b) and 2017 (Weller et al., 2020c) were used as the training and testing data, respectively, in the analyses reported here. Although the present study uses data from published empirical studies that characterized relationships between microbial water quality and environmental conditions, the study reported here is a survey focused on comparing algorithms and providing guidance for future modeling efforts.

**FIGURE 1:**
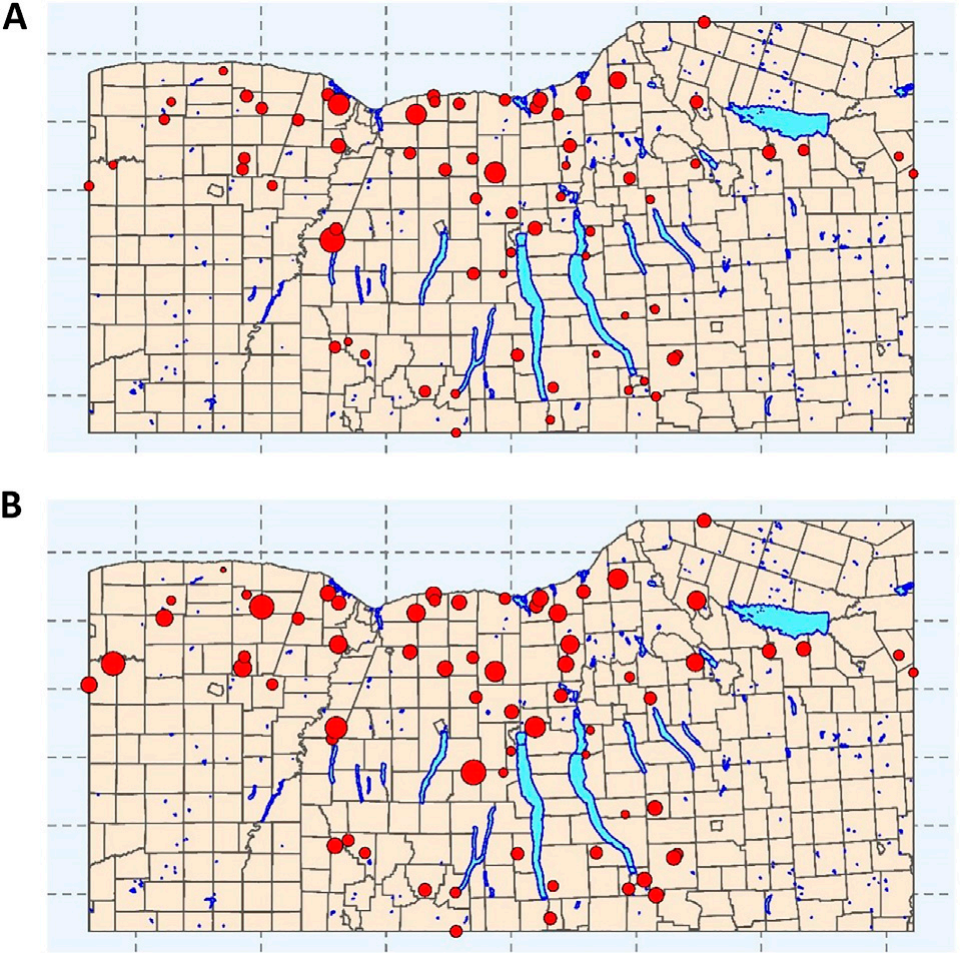
Sampling sites for each of the streams represented in the training and test data used here. Point size is proportional to **(A)** mean *E. coli* concentration (log10 MPN/100-ml) and **(B)** mean turbidity levels (log10 NTUs). Municipal boundaries (yellow), and major lakes (blue) are included as references. The map depicts the Finger Lakes, Western, and Southern Tier regions of New York State, United States.

Although the same sampling and laboratory protocols were used to generate both datasets, the datasets differ in the number of streams sampled (2017 = 6 streams; 2018 = 68 streams; Figure 1), and sampling frequency (2017 = 15–34 sampling visits per stream; 2018 = 2–3 visits per stream, (Weller et al., 2020b; Weller et al., 2020c)). As a result, the 2017 and 2018 data represent 181 and 194 samples, respectively, (Weller et al., 2020b; Weller et al., 2020c). At each sampling, a 1 L grab sample was collected and used for *E. coli* enumeration using the IDEXX Colilert-2000 test per manufacturer’s instructions (IDEXX, Westbrook, ME). Between sample collection and enumeration (<6 h), samples were kept at 4°C.

### Metadata

Spatial data were obtained from publicly available sources and analyzed using ArcGIS version 10.2 and R version 3.5.3. Briefly, the inverse-distance weighted (IDW) proportion of cropland, developed land, forest-wetland cover, open water, and pasture land for each watershed as well as the floodplain and stream corridor upstream of each sampling site was calculated as previously described [(King et al., 2005; Weller et al., 2020a; Supplementary Table S1). In addition to characterizing land cover, we also determined if specific features were present in each watershed. If a feature was present, the distance to the feature closest to the sampling site, and feature density were determined (for the full list see Supplementary Table S1).

Physicochemical water quality and air temperature were measured at sample collection (Weller et al., 2020c). Separately, rainfall, temperature, and solar radiation data were obtained from the NEWA weather station (newa.cornell.edu) closest to each sampling site (Mean Distance = 8.9 km). If a station malfunctioned, data from the next nearest station were used. Average air temperature and solar radiation, and total rainfall were calculated using non-overlapping periods (e.g., 0–1 day before sampling, 1–2 days before sampling; Supplementary Table S1).

### Statistical Analyses

All analyses were performed in R (version 3.5.3; R Core Team, Vienna, Austria) using the mlr package (Bischl et al., 2016). Model training and testing were performed using the 2018 (Weller et al., 2020b) and 2017 (Weller et al., 2020c) data, respectively. Hyperparameter tuning was performed using 3-fold cross-validation repeated 10 times. Tuning was performed to optimize root mean squared error (RMSE). After tuning, models were trained and performance assessed using RMSE, *R* ^2^, and Kendall’s Tau (*τ*). All covariates were centered and scaled before model development.

The algorithms used here were chosen to: 1) be comparable to algorithms used in past studies that predicted foodborne pathogen presence in farm environments [e.g., random forest, regression trees (Polat et al., 2019; Strawn et al., 2013; Golden et al., 2019; Weller et al., 2016)], and 2) include algorithms that appear promising but have not been previously utilized for produce safety applications (e.g., extremely randomized trees, cubist). Extensive feature engineering was not done before model implementation since the aim was to 1) compare algorithm performance on the same, unaltered dataset, and 2) as an opportunity to highlight where and how (e.g., for neural nets; Table 1) feature engineering may be needed. Moreover, due to the plethora of approaches to feature selection and engineering, a separate paper focused on assessing the impact of feature selection and engineering decisions on the performance of *E. coli* predictive models may be warranted. In total, 19 algorithms that fall into one of seven categories [support vector machines (SVM), cubist, decision trees, regression, neural nets, k-nearest neighbor (KNN), and forests] were used to develop the models presented here. However, a total of 26 models were developed using all predictors listed in Supplementary Table S1 (i.e., 26 full models) since multiple variations of the SVM (4 variations), cubist (4 variations), and KNN (2 variations) were considered. While advantages and disadvantages for each algorithm are outlined briefly in Table 1 and the discussion, more in-depth comparisons can be found in Kuhn and Johnson (Kuhn and Johnson, 2016).

**TABLE 1:** List of algorithms used in the study reported here. This table was adapted from Kuhn and Johnson (2016) and Weller et al., (2020a) to i) reflect the algorithms used here, and ii) report information relevant to continuous (as opposed to categorical) data^a^ ^,^ ^b^

Algorithm		Package	*n* < *p*	Centering and Scaling Recommended	For Features, It Can Handle				Automatic Feature Selection	Interpretable
					Correlation	Missingness	Near-Zero Variance	Noise		
Tree-based Learners										
	Conditional Inference Tree	party (Hothorn et al., 2006; Strobl et al., 2007a; Strobl et al., 2008; Strobl et al., 2009)	**Y**	**N**	**Y**	**Y**	**Y**	**•**	**Y**	**Y**
	Evolutionary Optimal Tree	evtree (Grubinger et al., 2014)	**Y**	**N**		**N**	**Y**	**•**	**Y**	**Y**
	Regression Tree^c^	rpart (Therneau and Atkinson, 2019)	**Y**	**N**	**•**	**Y**	**Y**	**•**	**Y**	**Y**
Ensemble Learners										
	Conditional Forest	party (Hothorn et al., 2006; Strobl et al., 2007a; Strobl et al., 2008; Strobl et al., 2009)	**Y**	**N**	**Y**	**•**	**Y**	**Y**	**•**	**•**
	Extremely Randomized Trees	extraTrees (Meinshausen, 2010)			**Y**	**Y**		**Y**	**Y**	
	Node Harvest^c^	nodeHarvest (Liaw et al., 2002)	**Y**	**N**	**•**	**Y**	**Y**	**Y**	**Y**	**•**
	Random Forest^c^	randomForest (Liaw et al., 2002)	**Y**	**N**	**•**	**Y**	**Y**	**Y**	**•**	**•**
	Regularized Random Forest	RRF (Deng and Runger, 2012; Deng and Runger, 2013)	**Y**	**N**	**Y**	**N**	**Y**	**Y**	**Y**	**•**
	Extreme Gradient Boosting	xgboost (Chen and Guestrin, 2016; Brownlee, 2019)	**Y**	**N**	**Y**	**Y**	**Y**	**Y**	**•**	**•**
Instance-Based Learners										
	k-Nearest Neighbor	kknn (Hechenbichler and Schliep, 2004)	**•**	**Y**	**N**	**N**	**N**	**•**	**N**	**N**
	Weighted k-Nearest Neighbor	kknn (Hechenbichler and Schliep, 2004)	**•**	**Y**	**N**	**N**	**N**	**•**	**N**	**N**
Multivariate Adaptive Regression Splines		earth (Milborrow, 2011)	**Y**	**N**	**Y**		**Y**	**•**	**Y**	**•**
Neural Network^d^		nnet (Venable et al., 2002)	**Y**	**N**	**N**		**N**	**N**	**N**	**N**
Regression										
	Log-Linear	stats	**N**	**Y**	**N**	**N**	**N**	**N**	**N**	**Y**
	Partial Least Squares	pls (Mevik et al., 2019)	**Y**	**N** ^e^	**Y**	**N**	**Y**	**N**	**•**	**Y**
	Principal Component	pls (Mevik et al., 2019)	**Y**	**N** ^e^	**Y**	**N**			**•**	
Penalized Regression										
	Elastic Net	glmnet (Friedman et al., 2010)	**Y**	**Y**	**Y**	**N**	**N**	**N**	**Y**	**Y**
	Lasso	glmnet (Friedman et al., 2010)	**Y**	**Y**	**Y**	**N**	**N**	**N**	**Y**	**Y**
	Ridge	glmnet (Friedman et al., 2010)	**N**	**Y**	**Y**	**N**	**N**	**N**	**N**	**Y**
Rule-Based Algorithms										
	Cubist	Cubist (Kuhn and Quinlan, 2018)	**Y**	**Y**	**Y**		**Y**	**Y**	**•**	**N**
SVM		e1071 (Meyer et al., 2020)	**Y**	**Y**	**•**	**N**	**Y**	**N**	**N**	**N**

^a^ The information reported here is based on i) Kuhn and Johnson, (2016), ii) the papers cited for each algorithm in the methods section, and iii) the constraints listed in the R packages below (based on the package version available in January 2020).

^b^ Y means the algorithm meets the condition in the header. N means the algorithm does not meet this condition. • means the algorithm is in between (e.g., random forest is not as interpretable as tree-based methods but is not a 100% black-box method like support vector machines). If the cell is blank it means there was limited information on this condition for the given algorithm.

^c^ Preferentially selects continuous factors and categorical factors with many levels as the splitting variable resulting in variable selection bias (Strobl et al., 2007b; Strobl et al., 2008; Strobl et al., 2009).

^d^ Feature selection recommended before model development.

^e^ Centering and scaling are required but are performed as part of model fitting in the R package.

**FIGURE 2:**
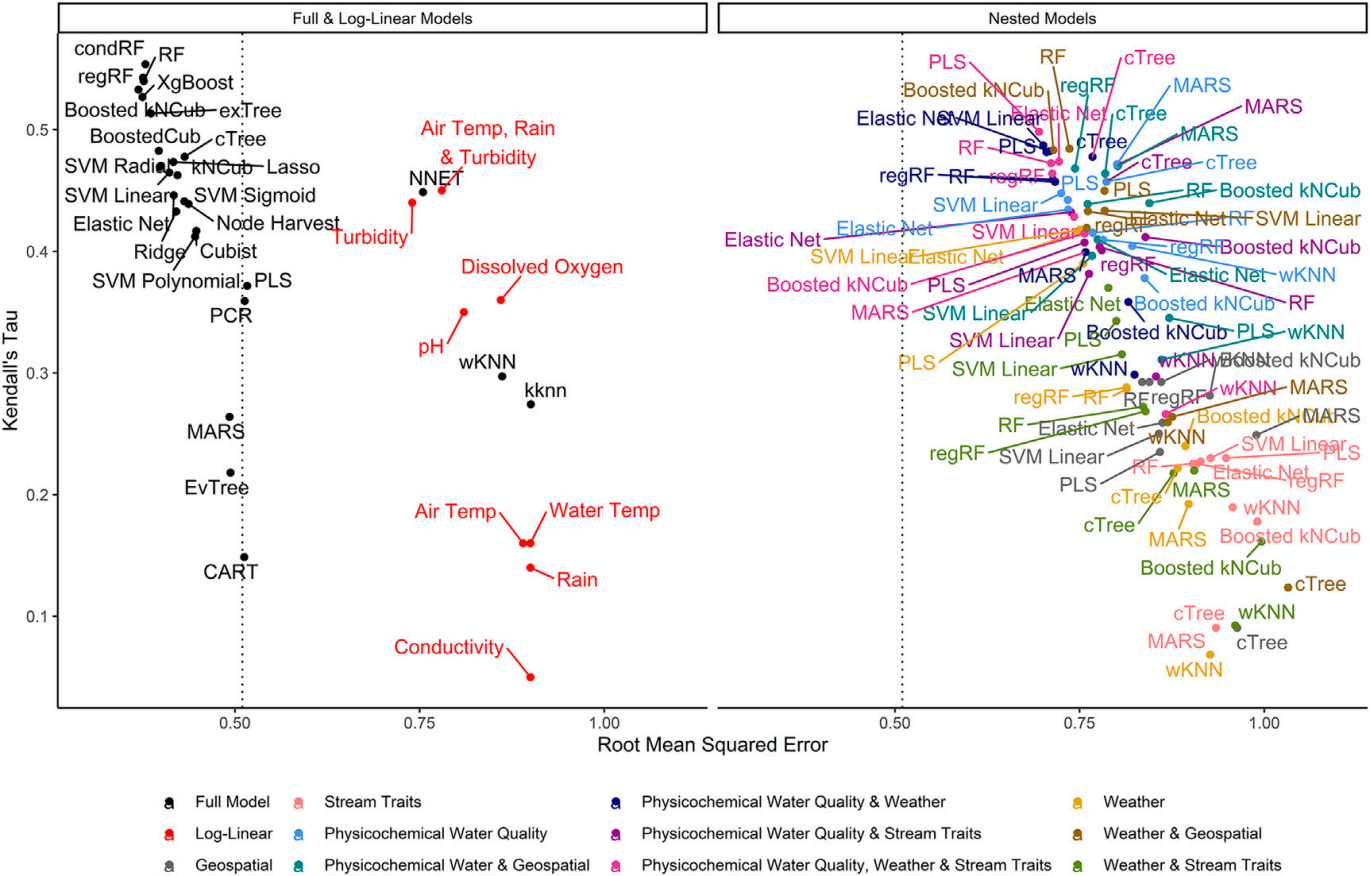
Graph of RMSE, which measures a model’s ability to predict absolute *E. coli* count, vs. Kendall’s tau, which measures the model’s ability to predict relative *E. coli* concentration. The dashed line represents the RMSE for the featureless regression model; an RMSE to the right of this line indicates that the model was unable to predict absolute *E. coli* counts. To facilitate readability, nested models are displayed in a separate facet from the full and log-linear models. Supplementary Figures S3, S4 display the nested model facet as a series of convex hulls to facilitate comparisons between models built using different feature types and algorithms, respectively. The top-performing models are in the top left of each facet.

Separately from the full models, nested models were built using different feature subsets (Supplementary Table S1). Features were divided into four categories: 1) geospatial, 2) physicochemical water quality and temperature data collected on-site, 3) all other weather data, and 4) stream traits that were observable on-site (e.g., composition of stream bottom). Nested models were built using different combinations of these four feature types, and one of nine algorithms (see Supplementary Table S3; Figure 2 for all feature-algorithm combinations; a total of 90 nested models). The nine algorithms used to build the nested models were randomly selected from the list of 26 algorithms used to build the full models.

Model performance was ranked using RMSE; models that tied were assigned the same rank. Two other performance measures, Kendall’s tau (*τ*) and the coefficient of determination (*R* ^2^) were also calculated. Kendall’s tau is a rank-based measure that assesses a model’s ability to correctly identify the relative (but not the absolute) concentration of *E. coli* in novel samples (e.g., if a sample was predicted to have a high or low *E. coli* concentration), while *R* ^2^ assesses how much variation in *E. coli* levels is predictable using the given model. Predictive performance for top-ranked models was visualized using density and split quantiles plots. An explanation of how to interpret these plots is included in the figure legends. For top-ranked models, the iml package (Fisher et al., 2018; Molnar et al., 2018) was used to calculate permutation variable importance (PVI) and identify the features most strongly associated with accurately predicting *E. coli* levels in the test and training data. Accumulated local effects plots were used to visualize the relationship between *E. coli* levels, and the six factors with the highest PVI (Apley and Zhu, 2016).

#### Baseline Models

Polat et al. (2019) developed a series of univariable models to predict *Salmonella* presence in Florida irrigation water. Each model was built using one of nine water quality or weather features (Polat et al., 2019). Studies conducted in nonagricultural, freshwater environments (e.g., swimming beaches) that focused on developing interpretable models used similar sets of physicochemical and weather features (Olyphant and Whitman, 2004; Francy and Darner, 2006; Efstratiou et al., 2009; Shiels and Guebert, 2010; Francy et al., 2013; Bradshaw et al., 2016; Dada, 2019). To ensure comparability with these previous studies, and provide baseline models that could be used to gauge full and nested model performance, we developed eight log-linear and a featureless regression model. Unlike the log-linear models, the featureless model did not include any features; models outperformed by the featureless model were unable to predict *E. coli* levels. Seven, separate univariable log-linear models were created using each of the following factors: air temperature at sample collection, conductivity, dissolved oxygen, pH, rainfall 0–1 day before sample collection, turbidity, and water temperature. An eighth model was built using air temperature, rainfall, and turbidity.

#### Tree-based and Forest Algorithm

Three tree-based algorithms were used: regression trees (CART), conditional inference trees (CTree), and evolutionary optimal trees (evTree) as described in a previous study focused on predicting pathogen presence in agricultural water (Weller et al., 2020a). Briefly, the three tree-based algorithms were implemented using the rpart (Therneau and Atkinson, 2019), party (Hothorn et al., 2006; Strobl et al., 2007b; Strobl et al., 2008; Strobl et al., 2009), and evtree (Grubinger et al., 2014) packages, respectively. The number of splits in each tree and the min number of observations allowed in terminal nodes were tuned for each algorithm. Complexity parameters were tuned to minimize overfitting when implementing the CART and evTree algorithms, while the mincriterion parameter was set to 0.95 when implementing the CTree algorithm.

Six ensemble algorithms [conditional forest (condRF); extreme gradient boosting (xgBoost); node Harvest; random forest (RF); regularized random forest (RRF), and exTree] were implemented. For the three random forest algorithms, the number of factors considered for each split, and the minimum number of observations allowed in terminal nodes was tuned. To minimize overfitting the coefficient of regularization was tuned for regRF models, while the mincriterion parameter was tuned for condRF models. When implementing the xgBoost algorithm (Chen and Guestrin, 2016), hyperparameters were tuned that control: 1) learning rate and overfitting; 2) if splits were formed and the max. number of splits allowed; 3) number of rounds of boosting; 4) proportion of data used to build each tree; 5) number of features considered when building each tree; and 6) regularization. When implementing the node Harvest algorithm, hyperparameters were tuned that control the: 1) min number of samples to use to build each tree, and 2) max. number of splits allowed in each tree. Unlike the five other forest-based learners, the number of samples used to build each tree was not tuned when implementing the exTree algorithm since neither bagging, bootstrapping, nor boosting is performed when building exTrees (Simm et al., 2014). Instead, hyperparameters were tuned that control the: 1) number of features considered when building each node; 2) the max. size of terminal nodes; and 3) the number of discretization points to select at random when defining a new node. The latter parameter highlights a key difference between the exTrees and random forest algorithms; random forests use local optimization to make the best split for a given node, which may not be globally optimal. To overcome this limitation and decrease computation time, both the variable used in new nodes, and the cutpoint used to split that variable were chosen randomly. For all ensemble methods the number of trees used was set to 20,001.

#### Instance-Based Algorithms

 Two instance-based algorithms [k-nearest neighbor (kKNN) and weighted k-nearest neighbor (wKNN)] were implemented (Hechenbichler and Schliep, 2004). Implementation of instance-based algorithms requires tuning the number of neighbors used when predicting a novel observation. Additionally, the method for calculating distances between neighbors (Euclidean or Manhattan) was tuned when implementing the KNN algorithms. For wKNN, the weighting kernel was also tuned since several weighting approaches exist.

#### Neural Nets

Neural networks are a non-linear regression technique and were implemented here using the mlr (Bischl et al., 2016) and nnet (Venable et al., 2002) packages. Unlike the other algorithms used here, neural nets cannot handle correlated or collinear predictors ((Kuhn and Johnson, 2016); Table 1). As such, feature selection was performed before fitting the neural nets by retaining all predictors with 1) non-zero coefficients according to the full elastic net model, and 2) non-zero variable importance measures according to the full condRF model. In neural net models, the outcome is predicted using an intermediary set of unobserved variables that are linear combinations of the original predictors (Kuhn and Johnson, 2016). As such, the number of intermediary variables used in the model was tuned as was the max. iterations run. Since neural net models often overfit (Kuhn and Johnson, 2016), a regularization parameter was also tuned.

#### Regression and Penalized Algorithms

Regression models, like the baseline models developed here, are frequently used to assess associations between features and food safety outcomes [e.g., (Wilkes et al., 2009; Benjamin et al., 2015; Ceuppens et al., 2015; Pang et al., 2017)]. However, conventional regression cannot handle correlated or collinear features or a large number of features. Various algorithms have been developed to overcome these limitations (Kuhn and Johnson, 2016). We used five such algorithms here, including penalized regression, partial least squares regression, and principal component regression. Penalized regression models apply a penalty to the sum of squared estimates of errors (SSE) to control the magnitude of the parameter estimates, and account for correlation between features (Kuhn and Johnson, 2016). All three penalized algorithms used here (ridge, lasso, and elastic net) were fit using the glmnet package using 10 cross-validated folds (Friedman et al., 2010), which automatically tunes lambda (amount of coefficient shrinkage). For all three models, a hyperparameter was tuned that determines if the model with the min mean cross-validated error or the model within one standard error of the min. was retained. For ridge and lasso regression, alpha was set to 0 or 1, respectively, while alpha was tuned for the elastic net model.

To overcome limitations associated with correlated features or having large numbers of features, principal components regression (PCR) uses a two-step approach. The dataset dimension is first reduced using principal components analysis (PCA), and then regression is performed using the principal components as features. Since PCA is performed independently of the outcome response, PCA may not produce components that explain the outcome, resulting in a poor-performing model (Kuhn and Johnson, 2016). Partial least squares regression (PLS) does not suffer from this limitation. Like PCR, PLS finds underlying, linear combinations of the predictors. Unlike PCR, which selects combinations to maximally summarize the features, PLS selects combinations that maximally summarize covariance in the outcome (Kuhn and Johnson, 2016). PCR and PLS models were both fit using the mlr (Bischl et al., 2016) and pls (Mevik et al., 2019) packages, and the number of components used was tuned. Since there are several variations of the PLS algorithm (Mevik et al., 2019), the PLS algorithm used was also tuned.

#### Multivariate Adaptive Regression Splines (MARS)

Like PLS and neural net, the MARS algorithm uses the features to create new, unobserved intermediary variables that are used to generate model predictions (Kuhn and Johnson, 2016). MARS creates each new intermediary using fewer features than PLS and neural net. MARS uses a piecewise linear regression approach that allows each intermediary to model a separate part of the training data and automatically accounts for interactions (Kuhn and Johnson, 2016). As in other approaches, once a full set of intermediaries has been created, pruning is performed to remove intermediaries that do not contribute to model performance (Kuhn and Johnson, 2016). The MARS models created here were implemented using the mlr (Bischl et al., 2016) and mda (Hastie and Tibshirani, 2017) packages. When fitting the MARS models the number and complexity of the intermediaries retained in the final model were tuned.

#### Rule-Based Algorithms

Four variations of the Cubist algorithm were implemented (Kuhn and Quinlan, 2018). Cubist models grow a tree where each terminal node contains a separate linear regression model. Predictions are made using these terminal models but smoothed using the model immediately above the given terminal node. Ultimately, this results in a series of hierarchical paths from the top to the bottom of the tree. To prevent overfitting these paths are converted to rules, which are pruned or combined based on an adjusted error rate (Kuhn and Johnson, 2016). Like tree-based models, an ensemble of Cubist models can be created and the predicted *E. coli* concentration from all constituent models averaged to obtain the model prediction (Kuhn and Quinlan, 2018). This version of the Cubist model is called boosted Cubist (BoostedCub). Separately, from BoostedCub, an instance-based Cubist can be used to create a k-nearest neighbor Cubist (kNCub (Kuhn and Quinlan, 2018)). kNCub works by first creating a tree, and averaging the prediction from the k-nearest training data points to predict the *E. coli* concentration in a novel sample (Kuhn and Quinlan, 2018). The BoostedCub and kNCub can also be combined to generate a boosted, k-nearest neighbor Cubist (Boosted kNCub (Kuhn and Quinlan, 2018)). For all Cubist models, the number of rules included in the final model was tuned. The number of trees used was tuned for the boosted Cubist models, and the number of neighbors used was tuned for the instance-based Cubist models.

#### Support Vector Machines

Four variations of support vector machines (SVM) were implemented using the e1071 package (Meyer et al., 2020). Each of the variations used a different kernel transformation. Each kernel mapped the data to higher or lower dimensional space, and the number of hyperparameters tuned reflects the dimensionality of the kernel. In order of most to least dimensionality, the kernels used were polynomial, sigmoidal, and radial; a linear kernel was also considered. Regardless of the kernel used, a penalty parameter that controls the smoothness of the hyperplane’s decision boundary was tuned. For all SVMs built using non-linear kernels, a parameter was tuned that determines how close a sample needs to be to the hyperplane to influence it. For the sigmoid and polynomial SVMs, a parameter that allows the hyperplane to be nonsymmetrical was tuned. For the polynomial SVM, the degree of the polynomial function was tuned.

## Results and Discussion

One-hundred twenty-five models were developed to predict *E. coli* levels in Upstate New York streams used for agricultural purposes (e.g., produce irrigation; Figure 2; 26 full models +90 nested models +9 baseline models). Full models were built using all four feature types, while nested models were built using between one and four feature types. The feature types considered were 1) geospatial, 2) physicochemical water quality and temperature data collected on-site, 3) all other weather data, and 4) stream traits observable on-site (e.g., stream bottom composition). Baseline models were either log-linear or featureless regression models.

The log_10_ MPN of *E. coli* per 100 ml was similarly distributed in the training (1st quartile = 1.95; median = 2.33; 3rd quartile = 2.73) and test data (1st quartile = 1.90; median = 2.21; 3rd quartile = 2.54). While an advantage of this study is the use of two independently collected datasets to separately train and test the models, the size of each dataset (N = 194 and 181 samples in the training and test data, respectively), as well as the temporal and geographic range represented (one growing season per dataset, one produce-growing region), are a limitation. However, this study’s aim was not to develop field-ready models; instead, this study provides a conceptual framework for how field-ready models can be built once multi-region and multi-year datasets are available.

By using a continuous outcome, the present study complements a recent publication that focused on binary, categorical outcomes [detection/non-detection of enteric pathogens (Weller et al., 2020a)]. To the authors’ knowledge, this is also the first study to compare the performance of models for predicting *E. coli* levels in agricultural water that were built using different feature types (i.e., geospatial, physicochemical water quality features, stream traits, and weather). Since the skill, capital, time, and computational power required to collect data on each feature type varies, the findings presented here will help future studies optimize data collection by focusing on key predictors (although other predictors may be important in other produce-growing regions). This in turn will help ensure that field-ready models developed as part of these future studies do not require growers to invest substantial time and money collecting multiple data types. For similar reasons (e.g., accessibility to growers, practicality for incorporating into on-farm management plans), future studies aimed at developing deployable models may want to consider the degree of feature engineering performed, however, such considerations were outside the scope of the present study.

### Trade-Offs Between Interpretability and Accuracy Need to be Considered When Selecting the Algorithm Used for Model Development

**FIGURE 3:**
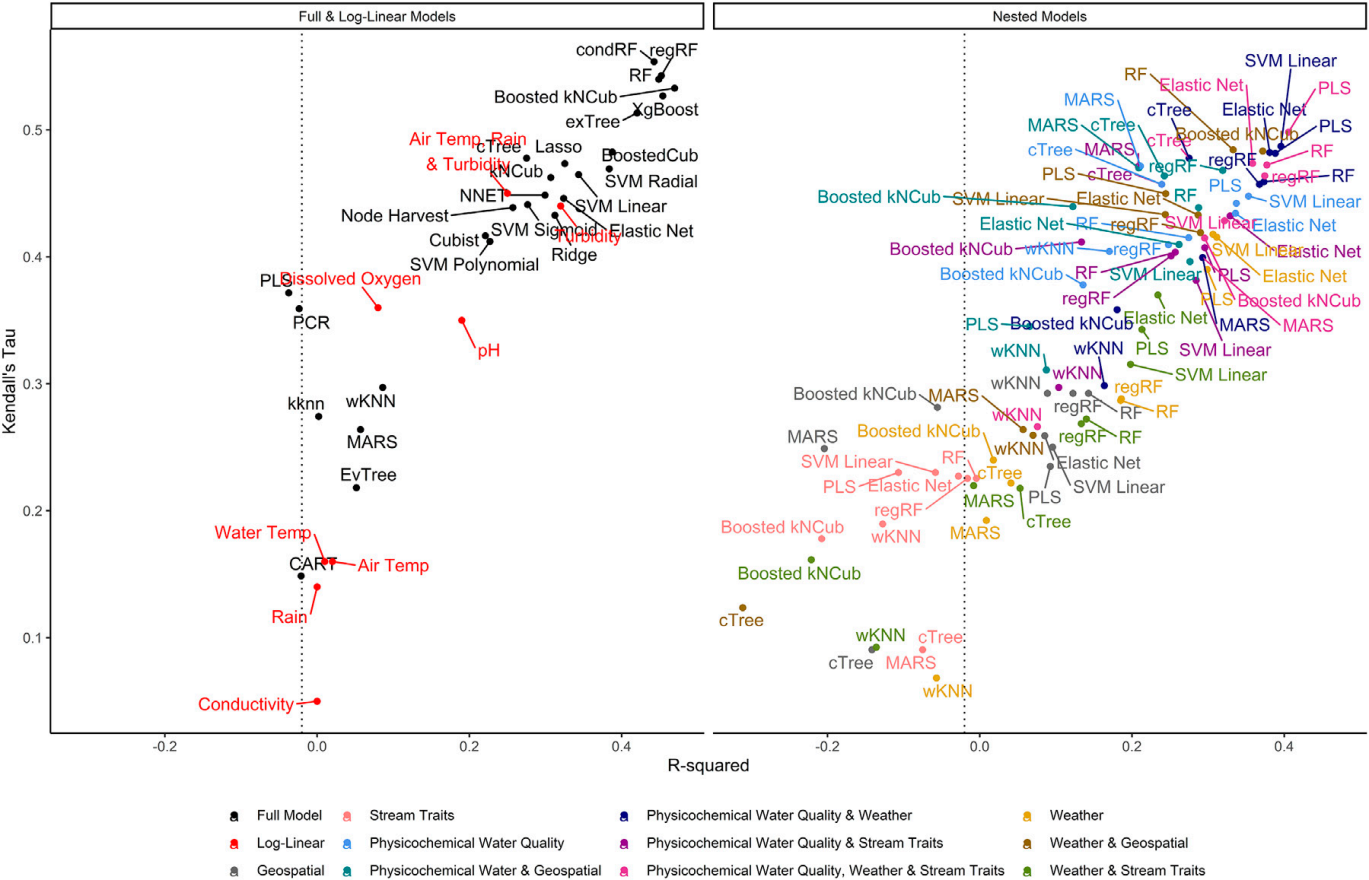
Graph of *R* ^2^, which measures the variance in *E. coli* levels that is predictable using the given model, vs. Kendall’s tau, which measures the model’s ability to predict relative *E. coli* concentration. The dashed line represents the *R* ^2^ for the featureless regression model; an *R* ^2^ to the left of this line indicates that the model was unable to predict variability in *E. coli* levels. To facilitate readability, nested models are displayed in a separate facet from the full and log-linear models. Better performing models are in the top right of each facet, while poor performers are in the bottom left.

**FIGURE 4:**
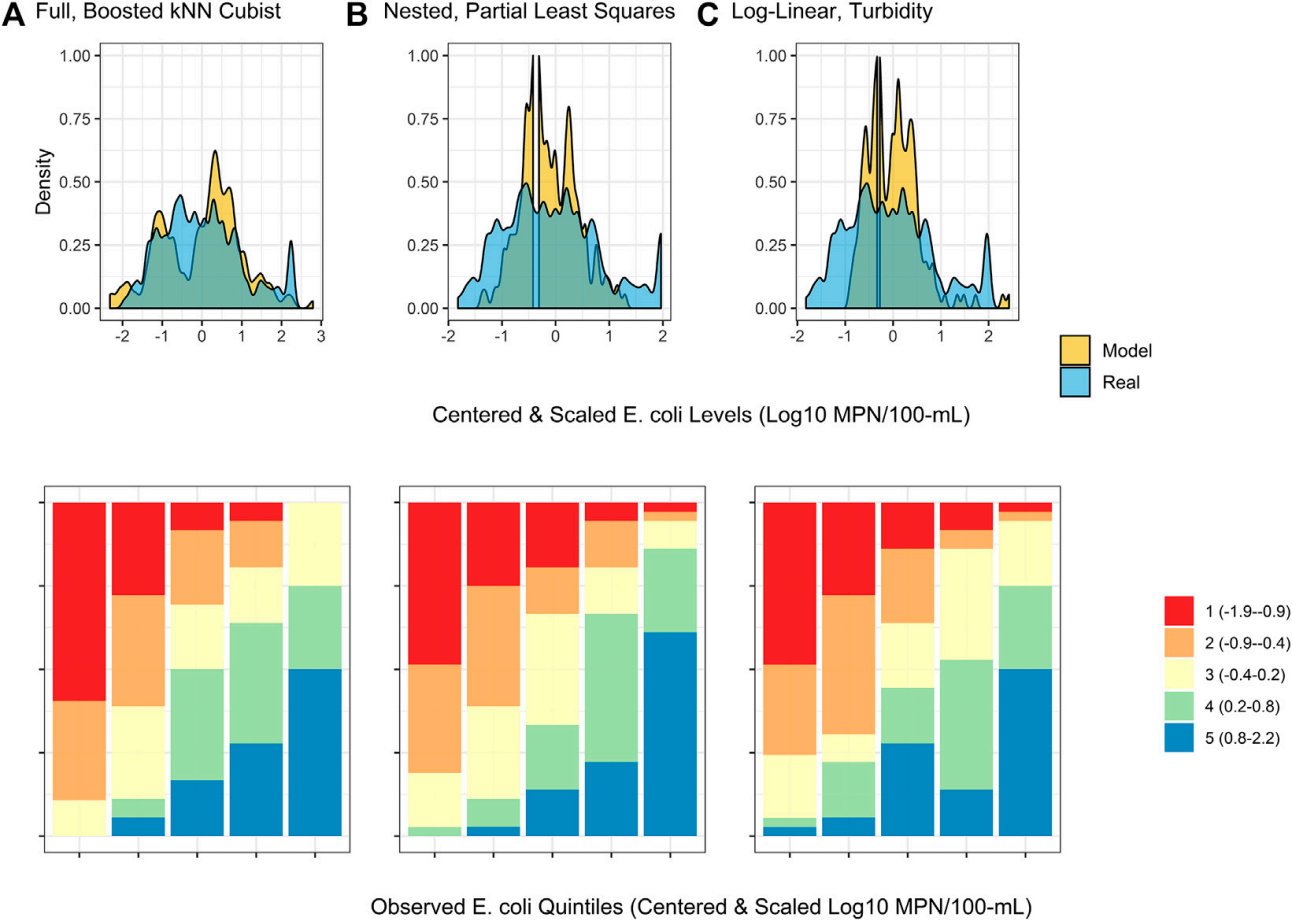
Density plots and split quantiles plots showing the performance of the top-ranked full, nested, and log-linear models. The density plot shows the ability of each model to predict *E. coli* counts in the test data, while the split quantiles plots show the ability of each model to predict when *E. coli* levels are likely to be high or low (i.e., relative *E. coli* concentration). The split quantiles plot is generated by sorting the test data from (i) lowest to highest predicted *E. coli* concentration, and (ii) lowest to highest observed *E. coli* concentration. The test data is then divided into quintiles based on the percentile the predicted value (color-coding; see legend) and observed values (*x*-axis goes from quintile with lowest observed *E. coli* levels on the left to highest on the right) fell into. In a good model, all samples that are predicted to have a low *E. coli* concentration (red) would be in the far left column, while samples that are predicted to have a high *E. coli* concentration (blue) would be in the far right column.

Model performance varied considerably with root-mean-squared errors (RMSE), Kendall’s Tau (*τ*), and *R* ^2^ ranging between 0.37 and 1.03, 0.07, and 0.55, and −0.31 and 0.47, respectively, (Supplementary Table S2; Figure 2). The top-performing full models all performed comparably and were built using either boosted or bagged algorithms. In order, the top-performing models were: Boosted kNCub (RMSE = 0.37); xgBoost (RMSE = 0.37); condRF (RMSE = 0.38); random forest (RMSE = 0.38); regRF (RMSE = 0.38); and exTree (RMSE = 0.39; Supplementary Table S2, Figures 2–4). These full models outperformed the top-ranked nested model, which was built using the PLS algorithm and water quality, weather, and stream trait factors (RMSE = 0.69; Supplementary Table S2, Figure 2). Below the cluster of best performing models in the top-left corner of Figure 2, there is a second cluster of models that performed well but not as well as the top-ranked models. It is interesting to note that the best-performing model in this second cluster was also built using a boosted algorithm (Boosted Cubist). Moreover, nested models built using ensemble algorithms generally outperformed those built using a tree or instance-based algorithm (Supplementary Figure S3). Overall, ensemble (boosted or bagged) algorithms substantially outperformed the other algorithms considered here. This is consistent with findings from a similar study (Weller et al., 2020a), which also found that ensemble models outperformed models built using alternative algorithms when predicting enteric pathogen presence in streams used to source irrigation water. These findings are also consistent with past studies that used ensemble methods (e.g., condRF, RF) to develop accurate models for predicting microbial contamination of recreational waters (Golden et al., 2019; Zhang et al., 2019; Munck et al., 2020) and agricultural environments (Golden et al., 2019).

Decision trees, which were previously proposed as candidate algorithms for developing interpretable, food safety decision-support tools (Magee, 2019), performed poorly here and in the aforementioned enteric pathogens study (Figure 2; Weller et al., 2020a). The poor performance of decision-trees is most likely due to overfitting during model training (Supplementary Figure S1). However, the fact that the RMSE of the interpretable models (e.g., tree-based models) was generally higher than the RMSE of black-box approaches (i.e., less interpretable models like SVM and ensemble algorithms) (Table 1; Figure 2), is illustrative of the trade-off between model interpretability and model performance [see (Meinshausen, 2010; Kuhn and Johnson, 2016; Doshi-Velez and Kim, 2017; Luo et al., 2019; Weller et al., 2020a) for more on these trade-offs]. Thus, our findings highlight the importance of weighing the need for interpretability vs. predictive accuracy before model fitting, particularly in future studies focused on developing implementable, field-ready models that growers can use for managing food safety hazards in agricultural water. When weighing these trade-offs, it is also important to consider that certain algorithms (e.g., conditional random forest) are better able to handle correlated and missing data as well as interactions between features than other algorithms (e.g., k-nearest neighbor, neural nets; Table 1). Similarly, it is important to consider whether feature selection is automatically performed as part of algorithm implementation (see Table 1). Since feature selection is performed as part of random forest implementation, they are more robust to the feature set used than neural nets or instance-based algorithms; this could explain the poor performance of the neural net, KNN, and wKNN models here. As such, random forest and similar algorithms may be able to better reflect the complexity and heterogeneity of freshwater systems particularly if feature selection will not be performed before model implementation.

### The Measure(s) Used to Assess and Compare Model Performance Should be Determined by How the Predictive Model Will be Used, and if Actual E. coli Counts or a Relative Concentration (i.e., High Versus Low) is Needed

It is important to highlight that only RMSE was used in hyperparameter tuning and to identify the best performing models. While RMSE-based rankings generally matched rankings based on *τ* and *R* ^2^, some models with high RMSE (which indicates worse performance) had similar *τ* and *R* ^2^ values to the top RMSE-ranked models (e.g., the neural net had high RMSE but *τ* and *R* ^2^ were similar to models with lower RMSE; Supplementary Table S2; Figure 2; Figure 4). This reflects differences in how each measure assesses performance. RMSE measures the differences between observed and predicted values, and therefore accounts for how off the prediction is from reality. As such, RMSE is a measure of how well the model can predict actual *E. coli* counts. Kendall’s *τ* is a rank-based measurement that does not account for absolute differences but instead ranks the observed data in order from highest to lowest value and determines how closely the predictions from the model match this ranking (Rosset et al., 2007). Thus, *τ* is useful for identifying models that can predict when *E. coli* levels are likely to be higher or lower (i.e., relative concentration) (Rosset et al., 2007). The coefficient of determination (*R* ^2^) reflects the proportion of variation in the outcome that is predictable by the model. In this context, our findings suggest that the neural net model is unable to predict actual *E. coli* concentrations but can correctly rank samples based on *E. coli* concentration (e.g., identify when levels are likely to be elevated). As such, neural nets may be appropriate for use in applied settings where the relative concentration but not the absolute count of *E. coli* is of interest (e.g., water source-specific models that are interested in deviation from baseline *E. coli* levels, which could indicate a potential contamination event). Indeed, a previous study that used neural nets to predict pathogen presence in Florida irrigation water was able to achieve classification accuracies (i.e., classify samples as having a high or low probability of contamination) of up to 75% (Polat et al., 2019). Conversely, neural nets may not be appropriate for predicting if a waterway complied with a water quality standard based on a binary *E. coli* cut-off. These results illustrate the importance of carefully considering how a model will be applied (e.g., are count predictions needed or are rank predictions needed, is interpretability or predictive accuracy more important) when selecting 1) the algorithm used for model fitting, and 2) the performance measure used for model tuning and assessing model performance (e.g., RMSE, *τ*, *R* ^2^).

The impact of performance measure choice on model interpretation and ranking is particularly clear when we examine the log-linear models developed here. The predictive accuracy of the log-linear models varied substantially. None of the variation in *E. coli* levels in the test data was predictable using the worst performing log-linear model (based on conductivity; RMSE = 0.90; *τ* = 0.05; *R* ^2^ = 0.0), while the best-performing log-linear model (based on turbidity) was able to predict 32% of the variation in the test data (RMSE = 0.74; *τ* = 0.44; *R* ^2^ = 0.32; Table S2). The performance of the turbidity model developed here is comparable to turbidity-based log-linear models developed to predict *E. coli* levels at Ohio swimming beaches (*R* ^2^ = 38% in (Francy and Darner, 2006); *R* ^2^ ranged between 19 and 56% in (Francy et al., 2013)). However, the RMSE of the turbidity model developed here was substantially worse than all full models except the wKNN and KNN models. Conversely, the *τ* and *R* ^2^ values of the turbidity model were comparable to or better than 11 of the full models (Supplementary Table S2). The only models to have substantially better *τ* and *R* ^2^ values than the turbidity model were models built using an ensemble algorithm (e.g., Boosted kNCub, regRF, RF, xgBoost; Supplementary Table S2). This suggests that the ability of the turbidity log-linear model to categorize the test data based on relative *E. coli* concentration (e.g., into samples with high or low predicted *E. coli* levels) was comparable to most full models. However, unlike the full models, the turbidity log-linear model could not predict actual *E. coli* concentrations in the test data samples. In fact, the density plots in Figure 4 graphically show how the top-ranked full model was substantially better at predicting *E. coli* counts compared to the top-ranked nested model and the turbidity log-linear model. Conversely, the split quantiles plots show that all three models were able to predict the relative concentration of *E. coli* in the test data samples (Figure 4). Overall, these findings reiterate the importance of determining how models will be applied in the field when designing a study. For example, if the aim is to develop an interpretable model to supplement ongoing monitoring efforts, a log-linear model based on turbidity could be useful for determining when *E. coli* concentration most likely deviates from baseline levels (e.g., are expected to be higher or lower). Such a model would be most useful if a baseline level of *E. coli* had been established for a given water source. However, separate models would need to be developed to establish this baseline for each water source, and the development of source-specific models could present an economic hurdle to small growers. As such, an ensemble model, like the full Boosted kNCub or XgBoost models developed here, would be more appropriate if 1) a generalized model (i.e., not specific to an individual water source): is needed, or 2) the model output needs to be an actual *E. coli* count.

### Accurate Predictions for Top-Ranked Models were Driven by Turbidity and Weather

**FIGURE 5:**
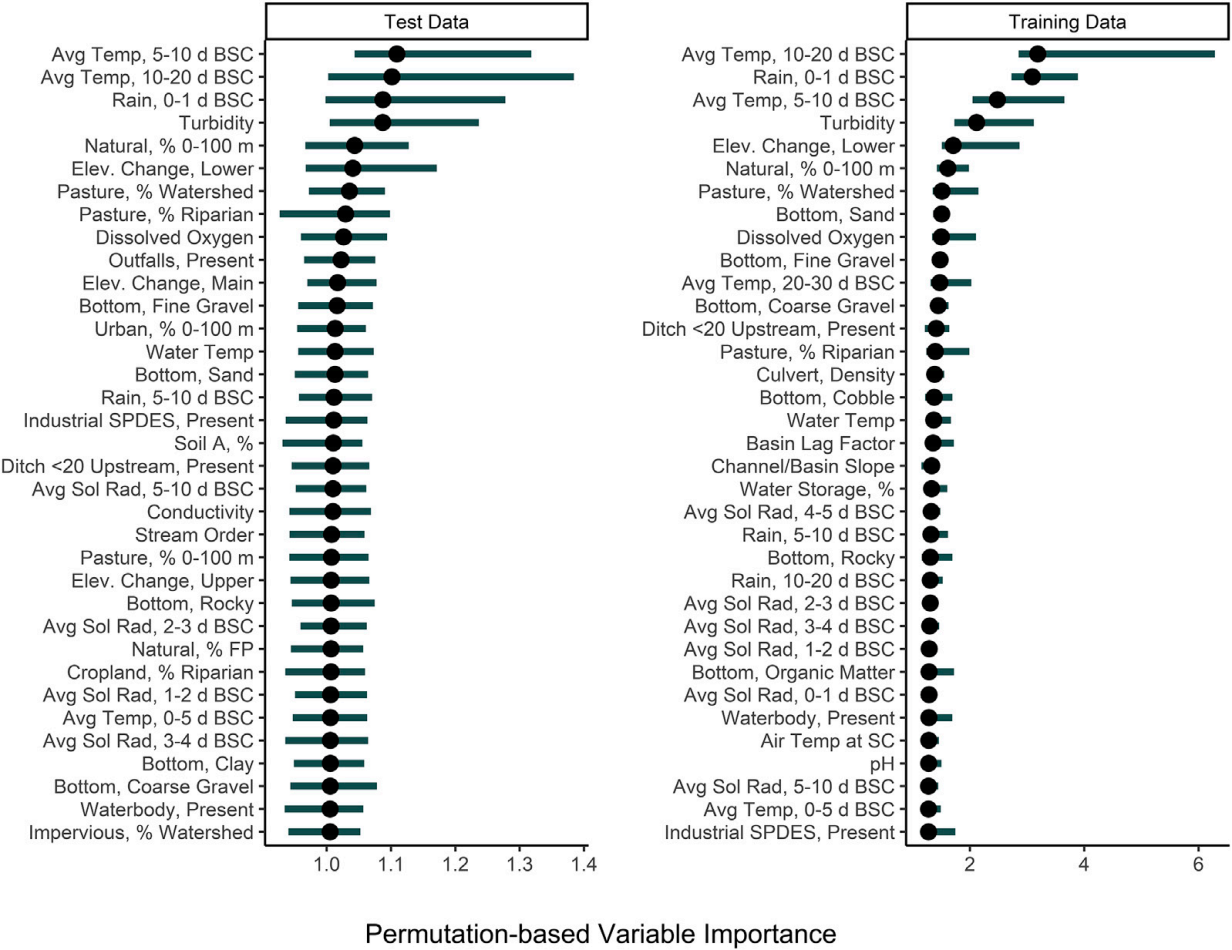
Permutation variable importance (PVI) of the 30 factors that were most strongly associated with predicting *E. coli* levels in the test and training data using full, boosted, k-nearest neighbor Cubist model. The black dot shows median importance, while the line shows the upper and lower 5% and 95% quantiles of PVI values from the 150 permutations performed. Avg. Sol Rad = Average Solar Radiation; Elev = Elevation; FP = Floodplain; SPDES = Wastewater Discharge Site; Soil A = Hydrologic Soil Type-A.

Among the nested models, models built using physicochemical and weather predictors consistently outperformed models built using geospatial predictors (Supplementary Table S2; Figure 2; Supplementary Figure S2). Indeed, by creating a convex hull graph that groups nested models by predictor type, the substantial differences in model performance due to predictor type are evident (Supplementary Figure S2). For example, all nested models built using only geospatial predictors or stream traits (e.g., stream bottom substrate) clustered in the bottom right of Supplementary Figures S2 (high RMSE, low *τ* indicating poor performance). For eight of the nine algorithms used to build the nested models, the full models had substantially lower RMSE values than the nested models, while nested models built using physicochemical water quality and/or weather features, on average, had substantially lower RMSE values than the geospatial nested models (Supplementary Table S2; Figure 2). However, since none of the nested models had an RMSE lower than the featureless regression, this indicates that all feature types (physiochemical, weather, geospatial, and stream traits) were needed to develop models that could accurately predict *E. coli* counts. That being said, many of the nested models had substantially higher *τ* and *R* ^2^ values than the featureless regression, indicating that they were able to accurately predict relative *E. coli* concentration (i.e., if it was higher or lower). The pattern observed for RMSE holds true for the *τ* and *R* ^2^ values, with physicochemical water quality models and weather models outperforming geospatial models. In fact, for several of the algorithms used for building the nested models, *τ* and *R* ^2^ values for the physiochemical and/or weather models were higher than *τ* and *R* ^2^ values for the full models, indicating that these models were better able to predict relative *E. coli* concentration than the full model. Based on permutation variable importance, the top-ranked full models’ ability to predict *E. coli* levels in both the training and test data was driven by air temperature, rainfall, and turbidity (Figure 5; Supplementary Figures S4, S5). Similarly, the top-ranked nested models’ ability to predict *E. coli* levels in the training and test data was driven by air temperature, rainfall, and turbidity, and by air temperature, solar radiation, and turbidity, respectively (Figure 5; Supplementary Figures S6–S8). Overall, these findings reiterate that appropriate features to use when training models for predicting *E. coli* levels in agricultural water source is dependent on how the model will be applied (i.e., if *E. coli* counts or relative concentration is needed). However, we can also conclude that regardless of how the model will be applied, physicochemical water quality and weather factors should be included as features and that geospatial features should not be used alone for model development. However, water quality is known to vary spatially (e.g., between produce-growing regions). Since this study was only conducted in one region, separate region-specific models or a single multi-region model may be needed.

**FIGURE 6:**
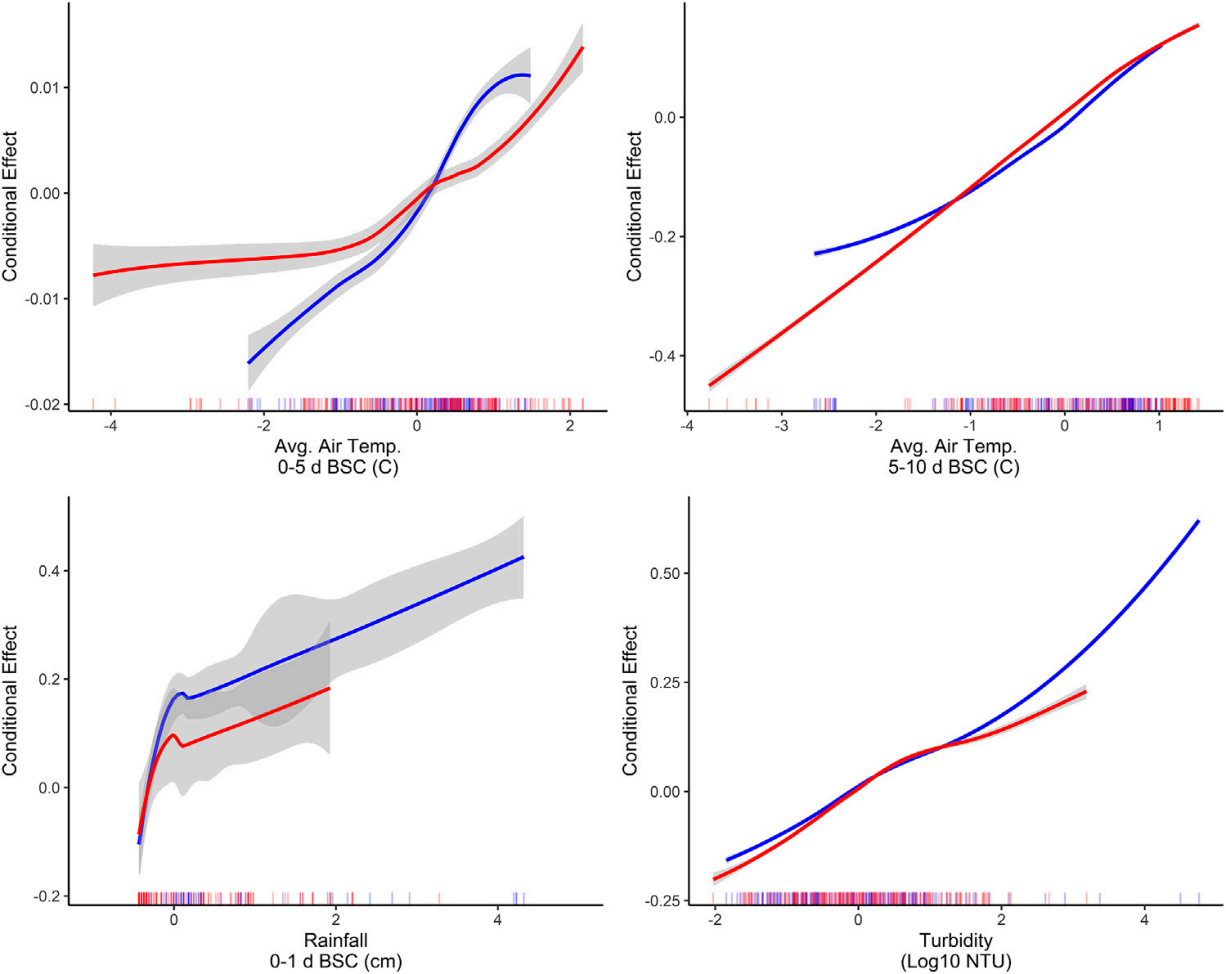
Accumulated local effects plots showing the effect of the four factors with the highest PVI when predictions were made on the training (shown in red) and test (shown in blue) data using the full boosted, k-nearest neighbor Cubist model. All predictors were centered and scaled before training each model. As a result, the units on the *x*-axis are the number of standard deviations above or below the mean for the given factor (e.g., in the rainfall plot 0 and two indicate the mean rainfall 0–1 day before sample are collection [BSC], and 2 standard deviations above this mean, respectively).

The identification of associations between microbial water quality, and physicochemical water quality, and weather features are consistent with the scientific literature (Francy et al., 2013; Bradshaw et al., 2016; Lawrence, 2012; Rao et al., 2015; Nagels et al., 2002; Liang et al., 2015). More specifically, the strong association between turbidity and *E. coli* levels, and rainfall and *E. coli* levels has been reported by studies conducted in multiple water types (e.g., streams, canals, recreational water, irrigation water, water in cattle troughs), regions (e.g., Northeast, Southeast, Southwest), and years, indicating that these relationships are reproducible even under varying conditions and when different study designs are used (Weller et al., 2020c; Brady et al., 2009; Brady and Plona, 2009; Francy and Darner, 2006; Lawrence, 2012; Smith et al., 2008; Davies-Colley et al., 2018; Olyphant et al., 2003; Money et al., 2009; Coulliette et al., 2009). For example, a study that sampled the Chattahoochee River, a recreational waterway in Georgia, United States of America, found that 78% of the variability in *E. coli* levels could be explained by a model that included log_10_ turbidity, flow event (i.e., base vs. stormflow), and season (Lawrence, 2012). In fact, the Georgia study found that for each log_10_ increase in turbidity *E. coli* levels increased by approx. 0.3 and approx. 0.8 log_10_ MPN/100-ml under baseflow and stormflow conditions, respectively (Lawrence, 2012). Similarly, in the study reported here, accumulated local effects plots indicate the presence of a strong, positive association between *E. coli* levels, and air temperature, rainfall, and turbidity (Figure 6). The fact that the *E. coli*-rainfall and *E. coli*-turbidity relationships are reproducible across studies, regions, and water types makes sense when viewed through the lens of bacterial fate and transport. Both rainfall and turbidity are associated with conditions that facilitate bacterial movement into and within freshwater systems (Nagels et al., 2002; Muirhead et al., 2004; Jamieson et al.,; Drummond et al., 2014). As such, it is not surprising that past studies that developed models to predict pathogen presence in agricultural water (Polat et al., 2019; Weller et al., 2020a) or *E. coli* concentrations in recreational water (e.g., (Francy and Darner, 2006; Brady et al., 2009; Brady and Plona, 2009)), found that models built using turbidity and/or rainfall outperformed models built using other factors. For example, Polat et al. (Polat et al., 2019) found that models that included turbidity as a feature were between 6 and 15% more accurate at predicting *Salmonella* presence in Florida irrigation ponds than models built using other predictors. Similarly, a study that used multivariable regression to predict *E. coli* level at Ohio beaches found that only rainfall and turbidity were retained in the final, best-performing model (Francy and Darner, 2006). Overall, the findings of this and other studies suggest that future data collection efforts (to generate data that can be used to train predictive *E. coli* models) should focus on physicochemical water quality and weather as opposed to geospatial factors.

While the scientific literature supports focusing future research efforts on collecting physicochemical water quality and weather data for models aimed at predicting *E. coli* presence or levels in the water, this recommendation is also supported by economic and computational feasibility. It is relatively easy and inexpensive for growers to obtain and download weather data from nearby extension-run weather stations since many growers already use these websites (e.g., NEWA [newa.cornell.edu], WeatherSTEM [www.weatherstem.com]) since this data is freely available. It can also be relatively inexpensive to collect turbidity and other physicochemical water quality data depending on the required precision of these measurements. Conversely, geospatial data requires either that: 1) the grower has access to software and training that allows them to extract geospatial data from government databases and calculate relevant statistics for each water source on their farm (e.g., the proportion of upstream watershed under natural cover), 2) an external group, such as consultants or universities working with industry perform this task, or 3) an external group develops a software program to perform this task. All three options would require substantial computational power, time, training, and capital.

## Conclusion

This study demonstrates that predictive models can be used to predict both relative (i.e., high vs. low) and absolute (i.e., counts) levels of *E. coli* in agricultural water in New York. More specifically, the findings reported here confirm previous studies’ conclusions that machine learning models may be useful for predicting when, where, and at what level fecal contamination (and associated food safety hazards) is likely to be in agricultural water sources (Polat et al., 2019; Weller et al., 2020a). This study also identifies specific algorithm-feature combinations (i.e., forest algorithms, and physicochemical water quality and weather features) that should be the foci of future efforts to develop deployable models that can guide on-farm decision-making. This study also highlights that the approach used to develop these field-ready models (i.e., the algorithm, performance measure, and predictors used) should be in how the model will be applied. For example, while ensemble methods can predict *E. coli* counts, interpretable (i.e., non-black-box methods like the baseline log-linear models) cannot. Conversely, these interpretable models were able to predict when *E. coli* levels are above or below a baseline. Overall, this proof-of-concept study provides foundational data that can be used to guide the design of future projects focused on developing field-ready models for predicting *E. coli* levels in agricultural and possibly other (e.g., recreational) waterways. Moreover, this paper highlights that accurate models can be developed using weather (e.g., rain, temperature) and physicochemical water quality (e.g., turbidity) features. Future efforts may want to focus on models built using these (as opposed to geospatial) features. In adapting these findings to guide the development of deployable models, it is important to note that several studies suggest that *E. coli* levels was an inconsistent predictor of pathogen presence in surface water (Harwood et al., 2005; McEgan et al., 2013; Pachepsky et al., 2015; Antaki et al., 2016; Weller et al., 2020c). Instead, *E. coli* models, like those developed here, may be useful for assessing fecal contamination status and for ensuring compliance with regulations but should not be used to determine if specific pathogens of concern (e.g., *Salmonella*, *Listeria*) are present.

## Data Availability

De-identified data (e.g., excluding GPS coordinates) are available on request. Requests to access these datasets should be directed to Daniel Weller, wellerd2@gmail.com/dlw263@cornell.edu.
