# Supplementary material for: Interpretability Versus Accuracy: A Comparison of Machine Learning Models Built Using Different Algorithms, Performance Measures, and Features to Predict E. coli Levels in Agricultural Water
Source: Front Artif Intell. 2021 May 14;4:628441. doi: 10.3389/frai.2021.628441 (PMC8160515; doi:10.3389/frai.2021.628441)
Supplement: Supplementary file 1 [file DataSheet1.docx]

Figure S1: Root mean squared error (RMSE) and Kendall’s tau for each full model when predictions were made on the training and test data, respectively. Lower RMSE and higher Kendall’s Tau estimates for predictions made on the training compared to test data indicates overfitting. Better performing models appear in the top left corner of each facet, while poor-performing models appear in the bottom left of each facet.


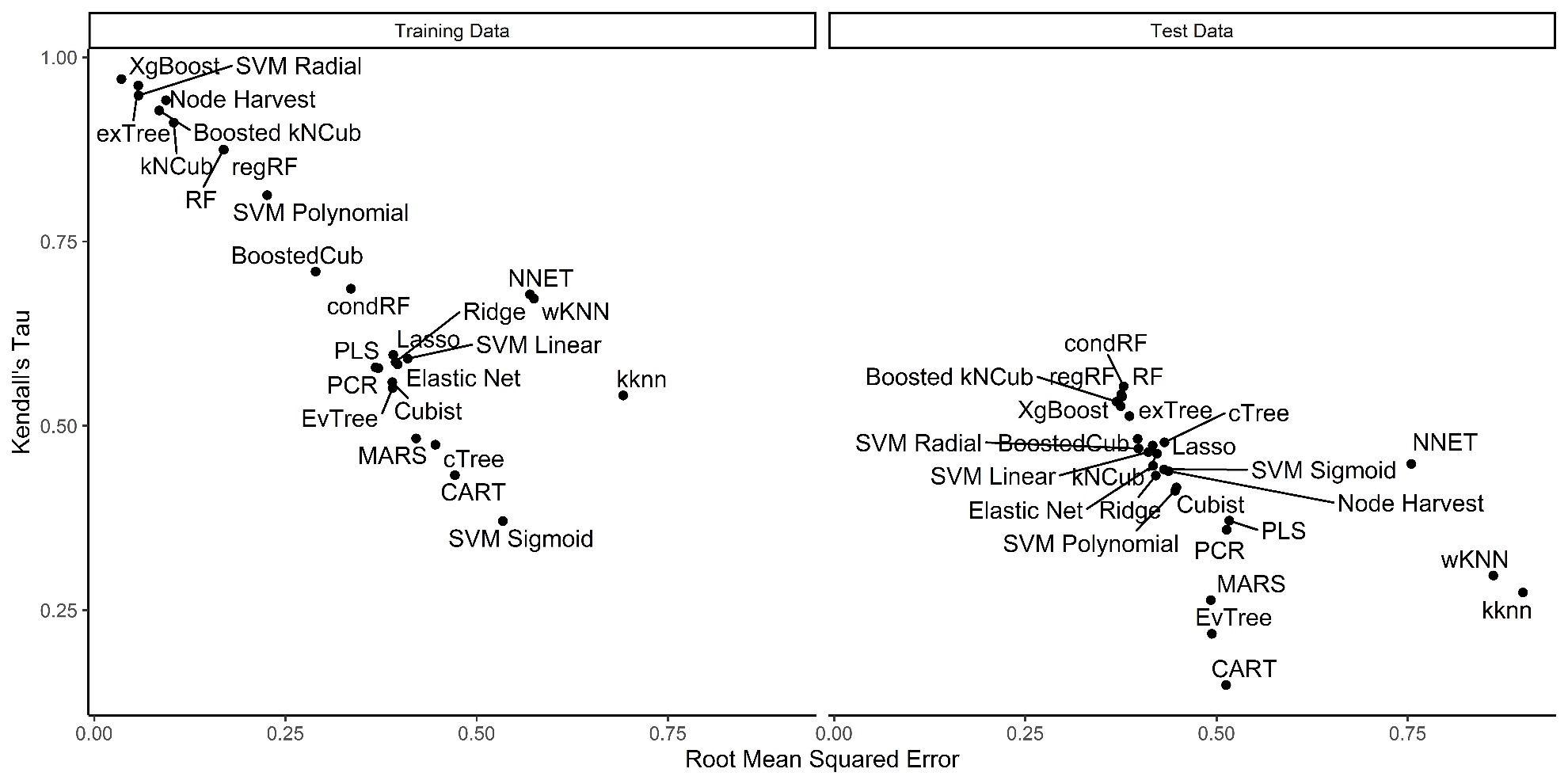


Figure S2: RMSE and Kendall's tau for each full and nested model built using the following algorithms: boosted k-nearest neighbor Cubist (Boosted kNCub), conditional inference tree (cTree), elastic net regression, multivariate adaptive regression splines (MARS), partial least squares (PLS), regularized random forest (regRF), random forest (RF), support vector machines with a linear kernel (SVM linear) and weighted k-nearest neighbor (wKNN). Better performing sets of models cluster in the top left corner of each facet, while poor-performing sets of models cluster in the bottom left of each facet.


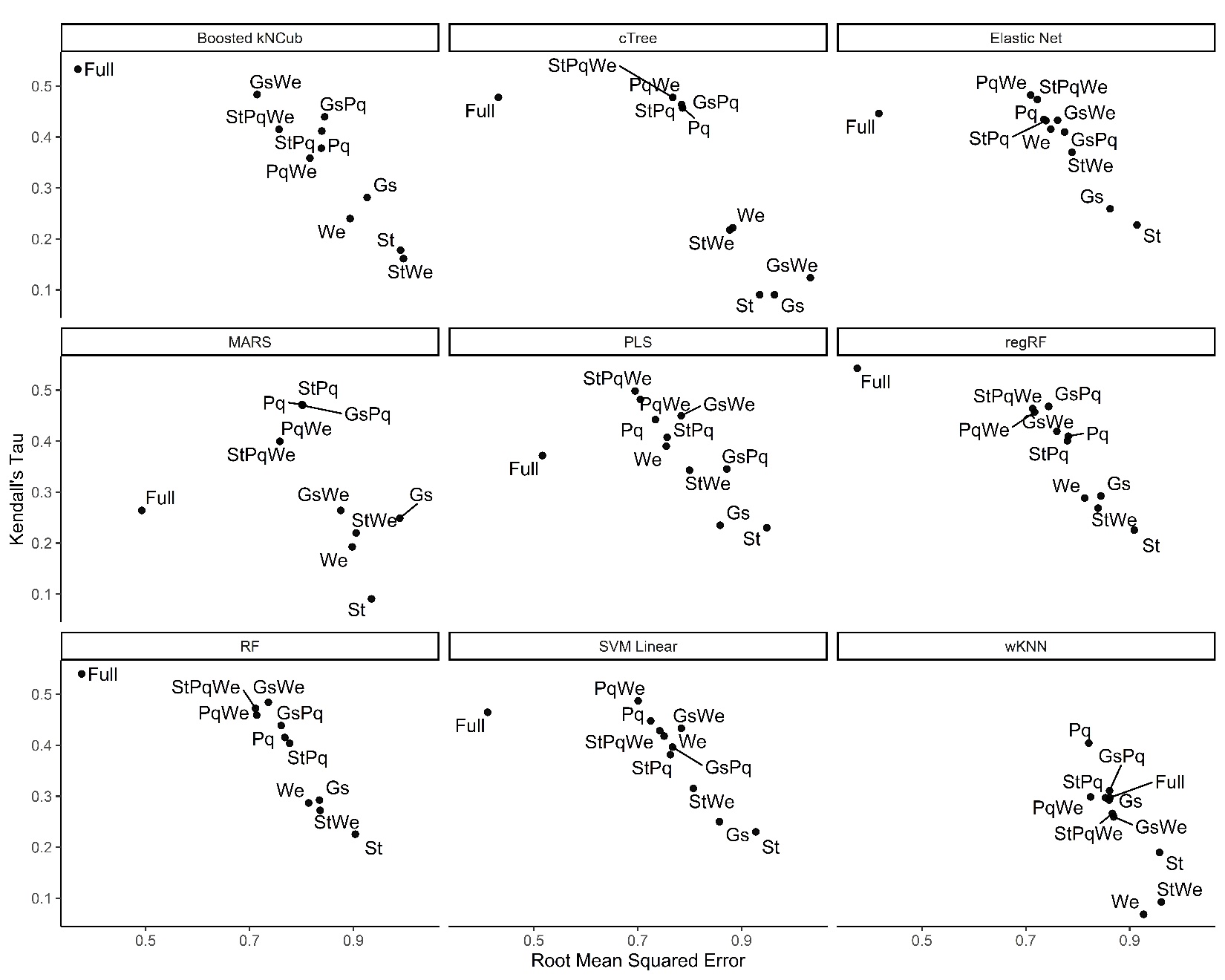


Figure S3: Convex hull showing how nested models built using each combination of predictor types cluster with regards to predictive performance on the test data. Better performing sets of models cluster in the top left corner of each facet, while poor-performing sets of models cluster in the bottom left of each facet.


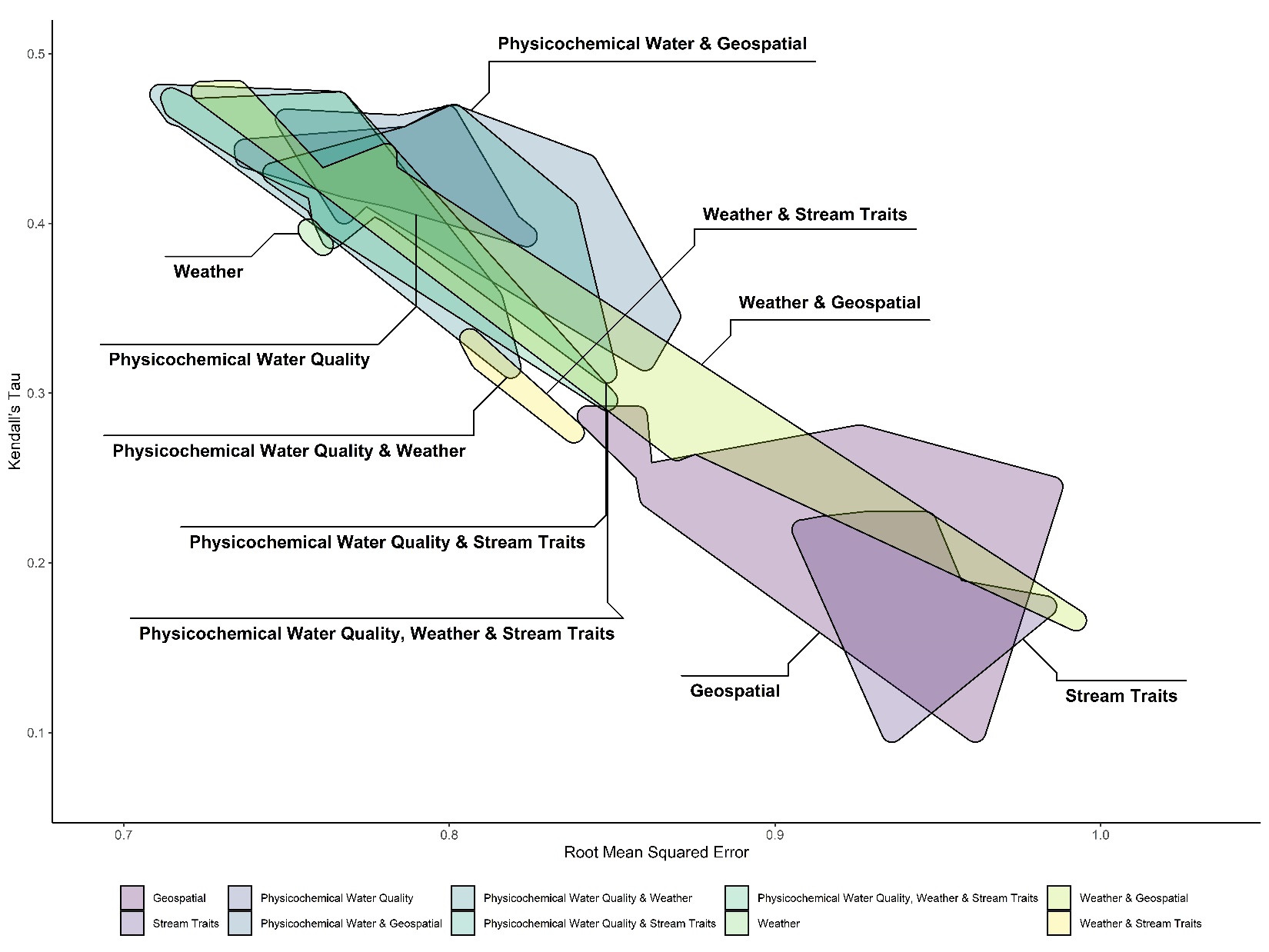


Figure S4: Convex hull showing how the nine algorithms used to develop the nested models cluster with regards to predictive performance on the test data. To facilitate readability and due to poor performance of nested models built using just geospatial data or just stream traits data across all nine algorithms, these models were not included in this figure. Better performing sets of models cluster in the top left corner of each facet, while poor-performing sets of models cluster in the bottom left of each facet.


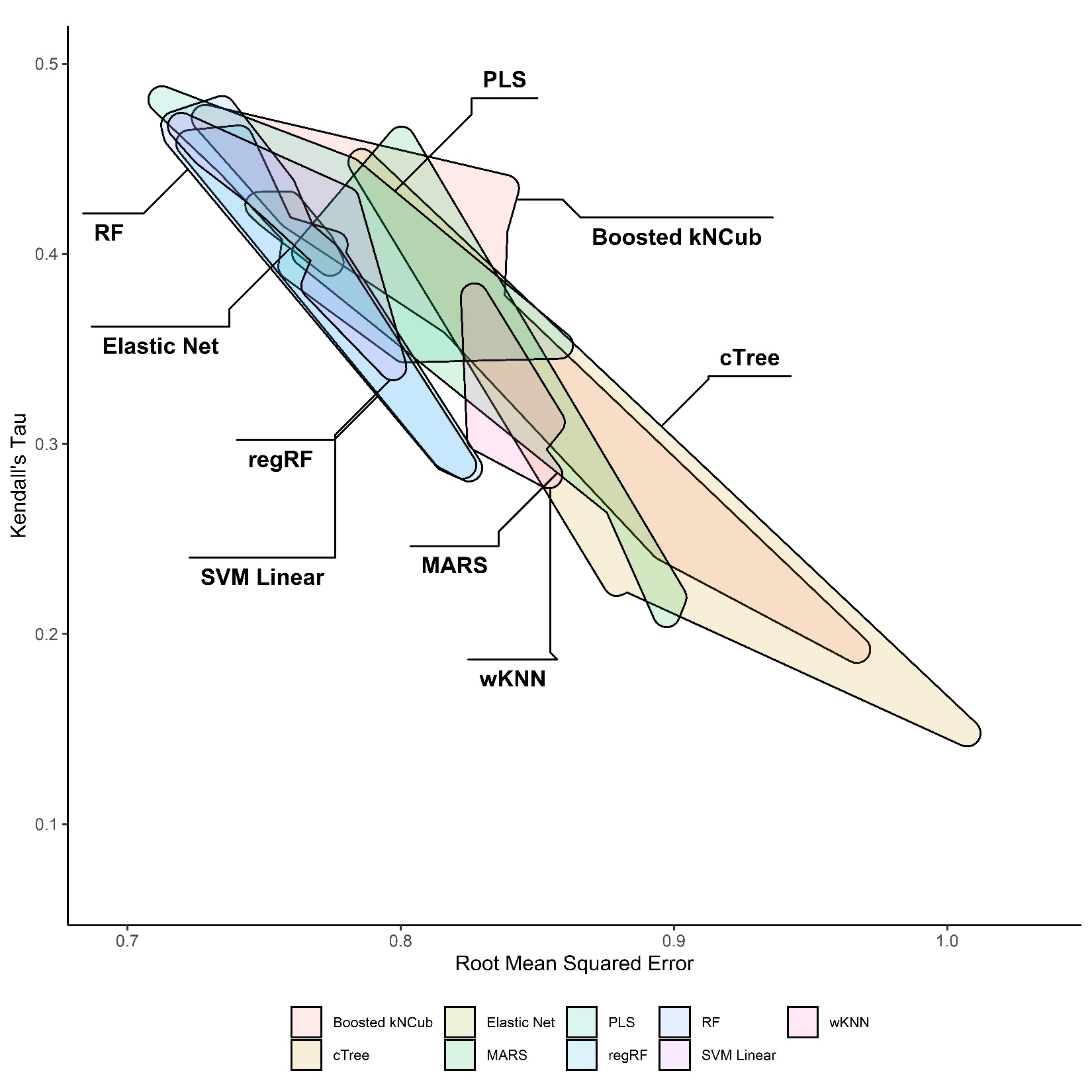


Figure S4: Permutation variable importance of the 30 factors that were most strongly associated with accurately predicting *E. coli* concentration in the test and training data using the full XgBoost model. The black point shows the median importance, and the green line shows the upper and lower 5% and 95% quantiles of importance values from the 150 permutations performed. Avg Sol Rad = Average Solar Radiation; Elev=elevation; FP=Floodplain; SPDES=wastewater discharge sites; Soil =Hydrologic Soil Type.


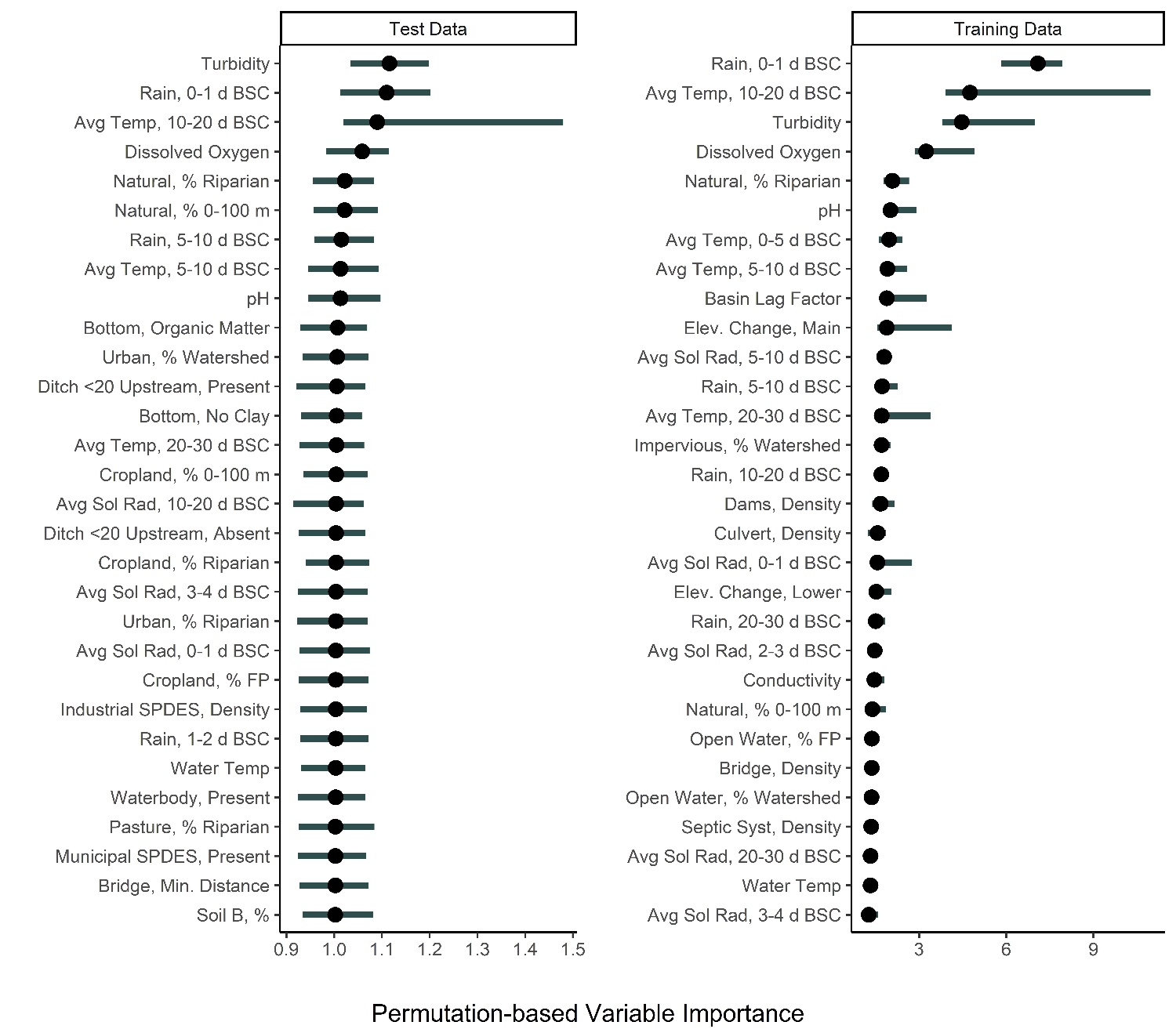


Figure S5: Permutation variable importance of the 30 factors that were most strongly associated with accurately predicting *E. coli* concentration in the test and training data using the full regularized random forest model. The black point shows the median importance, and the green line shows the upper and lower 5% and 95% quantiles of importance values from the 150 permutations performed. Avg Sol Rad = Average Solar Radiation; Elev=elevation; FP=Floodplain; SPDES=wastewater discharge sites; Soil=Hydrologic Soil Type.


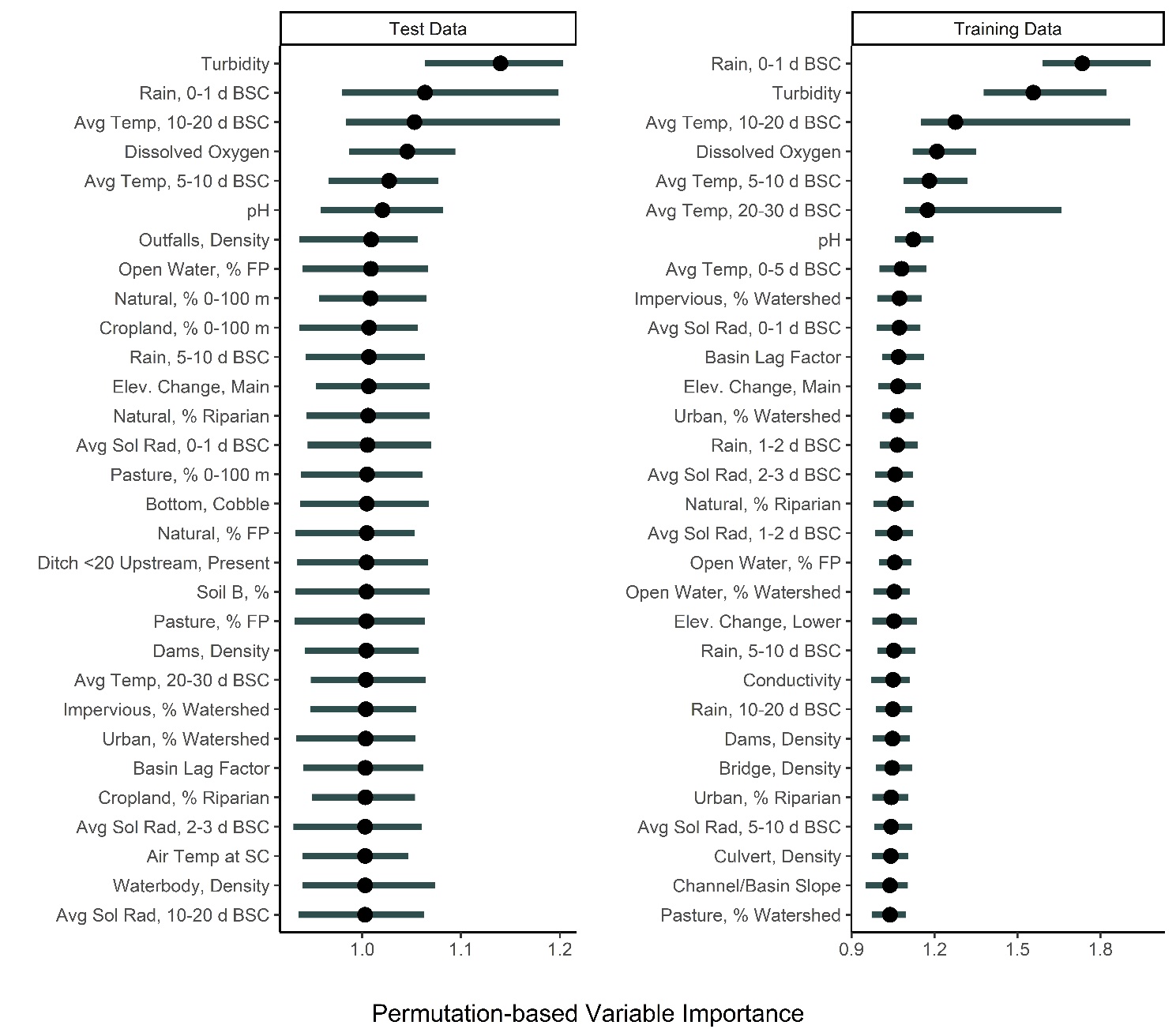


Figure S6: Permutation variable importance of the 30 factors that were most strongly associated with accurately predicting *E. coli* concentration in the test and training data using the partial least squares model and the physicochemical water quality, stream traits, and weather. The black point shows the median importance, and the green line shows the upper and lower 5% and 95% quantiles of importance values from the 150 permutations performed. Avg Sol Rad = Average Solar Radiation.


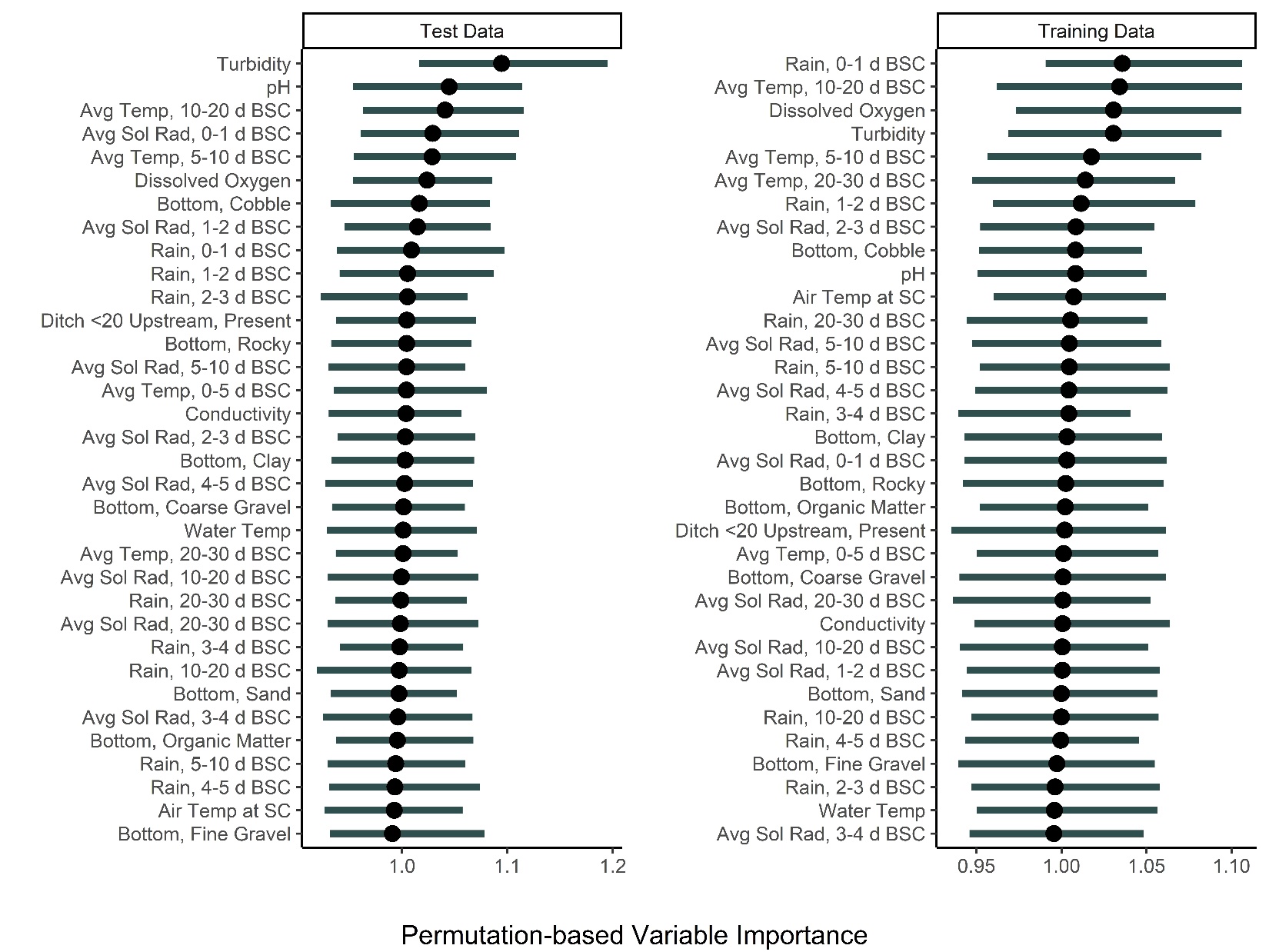


Figure S7: Permutation variable importance of the 30 factors that were most strongly associated with accurately predicting *E. coli* concentration in the test and training data using the support vector machine model and the physicochemical water quality, and weather factors. The black point shows the median importance, and the green line shows the upper and lower 5% and 95% quantiles of importance values from the 150 permutations performed. Avg. Sol Rad = Average Solar Radiation.


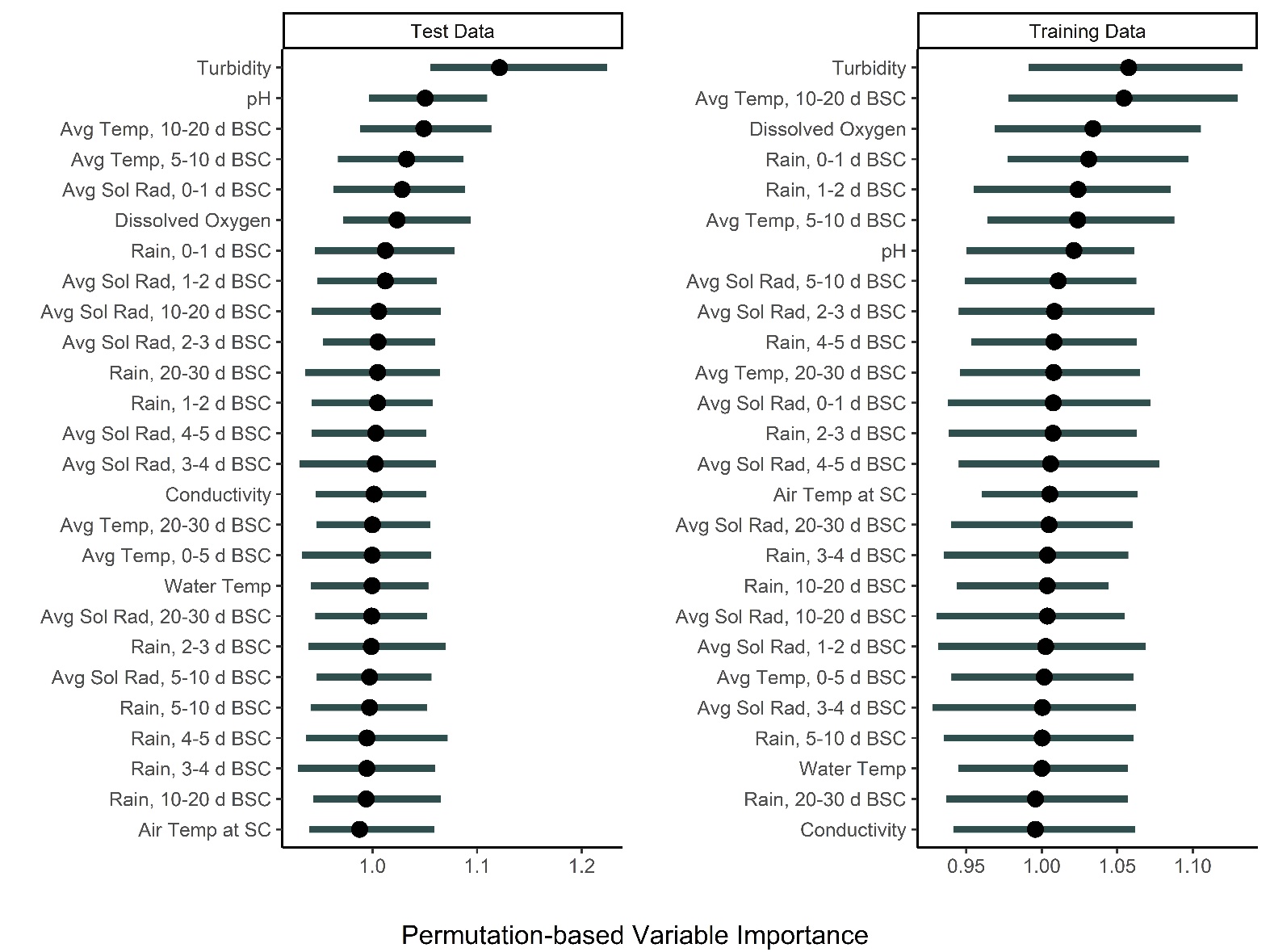


Figure S8: Permutation variable importance of the 30 factors that were most strongly associated with accurately predicting *E. coli* concentration in the test and training data using the partial least squares model and the physicochemical water quality, and weather factors. The black point shows the median importance, and the green line shows the upper and lower 5% and 95% quantiles of importance values from the 150 permutations performed. Avg. Sol Rad = Average Solar Radiation.


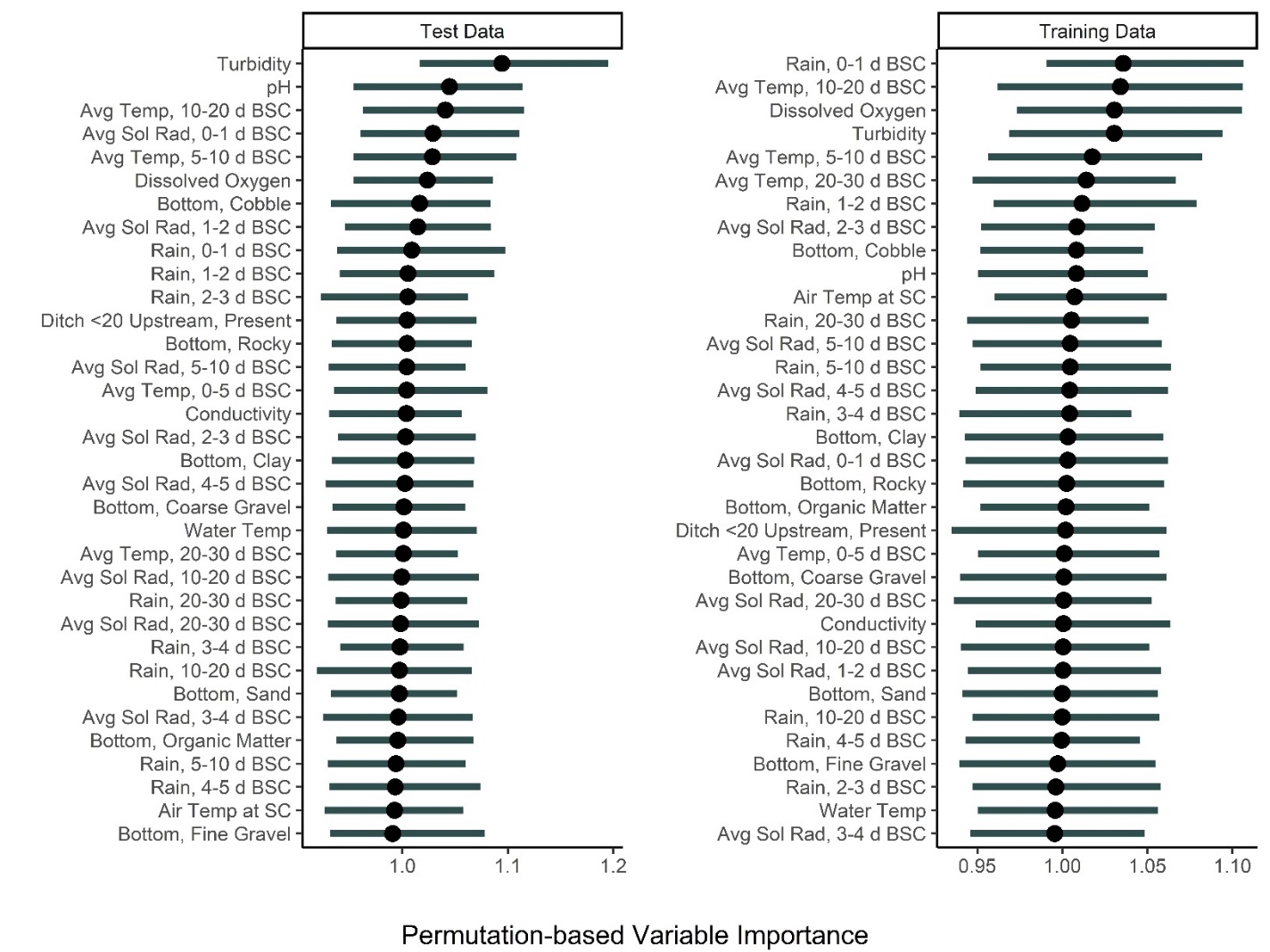


Table S1: Factors included in the analyses reported here. Values for all weather factors except for temperature were calculated for 0-1, 1-2, 2-3, 3-4, 4-5, 5-10, 10-20, and 20-30 d before sample collection (BSC). Values for temperature were calculated for 0-5, 5-10, 10-20, and 20-30 d BSC due to the strong correlation between temperature 0-1, 1-2, 2-3, 3-4, and 4-5 d BSC. This is a modified version of the table published in Weller et al. 2019 (Weller et al., 2020).

| Factor | | | | Description | Data Type ^a^ | Date | Citations and Websites |  |
| --- | --- | --- | --- | --- | --- | --- | --- | --- |
| Data Extracted from Publicly-Available Databases | | | | |  |  |  |  |
|  | Culverts: Carrying a waterway under a state highway and have a span of 5 to 20 feet | | | |  | 2014 | (NYS Large Culverts; Bridge Inventory Manual, 2006; Culvert inventory and inspection manual, 2006) |  |
|  |  | Present | | Were culverts present upstream? | Gs |  |  |  |
|  |  | Density | | Upstream density (no. of per 10 km^2^) | Gs |  |  |  |
|  | Dams | | |  |  | 2018 | (Dams) |  |
|  |  | Density | | Upstream density (no. of per 10 km^2^) | Gs |  |  |  |
|  | Road Crossing | | |  |  | 2015 | (USGS National Transportation Dataset) |  |
|  |  | | Density | Upstream density (no. of per 10 km^2^) | Gs |  |  |  |
|  |  | | Min. Dist. | The flow path distance to the nearest point upstream where a road crossed the stream (km). | Gs |  |  |  |
|  | Outfalls: Municipal stormwater outfalls along or near highways (presence is an indicator of urbanization) | | | |  | 2008 | (Outfall and system mapping for illicit discharge detection and elimination (IDDE) in NY; Methodology for the identification and survey of stormwater outfalls within designated MS4 locations for New York State DOT, 2004; Guidance on outfall mapping: What is an outfall, and what should be mapped?, 2006) |  |
|  |  | Present | | Were stormwater outfalls present upstream? | Gs |  |  |  |
|  |  | Density | | Upstream density (no. of per 10 km^2^) | Gs |  |  |  |
|  | Municipal Wastewater Discharge Sites: Based on permits issued under the NYS^a^ Pollutant Discharge Elimination System (SPDES) | | | |  |  |  |  |
|  |  | Present | | Were wastewater discharge sites present upstream? | Gs |  |  |  |
|  |  | Density | | Upstream density (no. of per 10 km^2^) | Gs |  |  |  |
|  | Industrial Wastewater Discharge Sites: Based on permits issued under the NYS^b^ Pollutant Discharge Elimination System (SPDES) | | | |  | 2018 | (State Pollutant Discharge Elimination System) |  |
|  |  | Present | | Were wastewater discharge sites present upstream? |  |  |  |  |
|  |  | Density | | Upstream density (no. of per 10 km^2^) |  |  |  |  |
|  | In-stream Waterbodies: Bodies of water within the stream channel (e.g., mill ponds, impoundments, lakes) | | | |  | 2017 | (National Hydrography Database) |  |
|  |  | Present | | Were waterbodies upstream? | Gs |  |  |  |
|  |  | Density | | Upstream density (no. of per 10 km^2^) | Gs |  |  |  |
|  | Septic System Density | | | Upstream density (no. per 10 km^2^). | Gs | 2011 | (Septic Systems, New York State, 2011 - CUGIR) |  |
|  | Solid Waste Site: Based on permits issued by NYS that allow application and spreading of manure, human septage, food processing, or other waste | | | |  | 2019 | (Solid Waste Management Facilities \| Open Data NY) |  |
|  |  | Density | | Upstream density (no. of per 10 km^2^) | Gs |  |  |  |
|  | Land Cover ^c^ | | | |  | 2016 | (NLCD 2016 Land Cover (CONUS), 2019; NLCD 2016 Percent Developed Imperviousness, 2019) |  |
|  |  | Open Water | | Class 11 in National Land Cover Database (NLCD) | Gs |  |  |  |
|  |  | Cropland | | Cropland; Class 82 in NLCD | Gs |  |  |  |
|  |  | Pasture | | Pasture; Class 81 in NLCD | Gs |  |  |  |
|  |  | Developed | | Developed; Classes 21-24 in NLCD | Gs |  |  |  |
|  |  | For-Wet | | Natural cover; Classes 41-43, 51-52, 90, and 95 in NLCD (i.e., forest, shrubland, or wetland) | Gs |  |  |  |
|  |  | Impervious | | Percent of upstream watershed that was under impervious cover | Gs |  |  |  |
|  | Watershed Area | | | Total area of upstream watershed (10-km^2^) | Gs | - | - |  |
|  | Stream Stats Data | | |  |  | 2019 | [streamstats.usgs.gov/ss/](https://streamstats.usgs.gov/ss/) |  |
|  |  | Avg. Basin Slope | | Mean basin lope determined by summing lengths of all contours in basin, multiplying by contour interval, and dividing product by drainage area | Gs |  |  |  |
|  |  | Elev. Contours, Length | | Total length of all elevation contours in drainage area in miles | Gs |  |  |  |
|  |  | Elev. Change, Main | | Change in elevation divided by length between points 10 and 85 percent of distance along main channel to basin divide | Gs |  |  |  |
|  |  | Elev. Change, Lower | | Change in elevation between points 10 and 85 percent of length along the lower half of the main flow path divided by length between the points | Gs |  |  |  |
|  |  | Elev. Change, Upper | | Change in elevation between points 10 and 85 percent of length along the upper half of the main flow path divided by length between the points | Gs |  |  |  |
|  |  | Elev. >1,200 ft, % | | Percentage of basin at or above 1200 ft elevation | Gs |  |  |  |
|  |  | Basin Lag Factor | | Basin Lag factor as defined in SIR 2006-5112 | Gs |  |  |  |
|  |  | Main Channel, Length | | Length along the main channel from the measuring location extended to the basin divide | Gs |  |  |  |
|  |  | Channel/Basin Slope | | Ratio of main channel slope to basin slope | Gs |  |  |  |
|  |  | Soil A, % | | Percentage of area of Hydrologic Soils Type A from SSURGO | Gs |  |  |  |
|  |  | Soil B, % | | Percentage of area of Hydrologic Soils Type B from SSURGO | Gs |  |  |  |
|  |  | Water Storage, % | | Percentage of area of storage (lake, ponds, reservoirs, wetlands) | Gs |  |  |  |
|  |  | Stream Level | | Stream level | Gs |  |  |  |
|  |  | Stream Order | | Strahler stream order | Gs |  |  |  |
|  |  | All Channels, Length | | An estimate of miles of stream upstream of a flowline | Gs |  |  |  |
| Water Quality and Other Conditions at Time of Sample Collection | | | | |  |  |  |  |
|  | Air Temp. at site | | | Air temperature measured at the sampling site at the time of sample collection (°C) | Pq | - | - |  |
|  | Conductivity | | | Conductivity (Log_10_ uS/cm) | Pq | - | - |  |
|  | Dissolved oxygen | | | Dissolved oxygen levels (mg/L) | Pq | - | - |  |
|  | Flow rate | | | Flow rate measured 3-6” below the surface (m/s) | Pq | - | - |  |
|  | pH | | | pH | Pq | - | - |  |
|  | Turbidity | | | Turbidity (Log_10_ NTU) | Pq | - | - |  |
|  | Water Temp. | | | Water temperature (°C) | Pq | - | - |  |
| Field-Collected Site Data | | | | |  |  |  |  |
|  | Ditch | | | Did a roadside ditch intersect the stream < 20 m upstream of the sample site? | St | ~~-~~ | ~~-~~ |  |
|  | Bottom Substrate: Composition of the stream bottom in the reach 10 m upstream of the sample site. The different categories of substrate were boulder, bedrock, cobble or larger, coarse gravel, fine gravel, sand, clay, and organic matter. | | | |  | ~~-~~ | (Unified Stream Assessment: A User’s Manual, 2004) |  |
|  |  | Rocky | | Was the substrate that comprised the majority of the bottom rocky (bedrock, boulder, cobble, or gravel) or not rocky (sand, clay, or organic matter/silt)? | St | - | - |  |
|  |  | Sand | | Was sand present along the stream bottom? | St | - | - |  |
|  |  | Clay | | Was clay present along the stream bottom? | St | - | - |  |
|  |  | Organic Matter | | Was organic matter present along the stream bottom? | St | - | - |  |
|  |  | Cobble or Larger | | Were cobble, boulders, or bedrock along the stream bottom? | St | - | - |  |
|  |  | Fine gravel | | Was fine gravel present along the stream bottom? | St | - | - |  |
|  |  | Coarse gravel | | Was coarse gravel present along the stream bottom? | St | - | - |  |
| Weather Data from NEWA Weather Stations | | | | |  |  |  |  |
|  | Avg. Air Temp. | | | Average temperature (°C) either 0-5, 5-10, 10-20 or 20-30 d before sample collection | W | - | [newa.cornell.edu](http://newa.cornell.edu/) |  |
|  | Avg. Solar Radiation | | | Average solar radiation (MJ/m^2^) either 0-1,1-2, 2-3, 3-4, 4-5, 5-10, 10-20 or 20-30 d before sample collection | W | - | [newa.cornell.edu](http://newa.cornell.edu/) |  |
|  | Total rainfall | | | Total rainfall (cm) either 0-1,1-2, 2-3, 3-4, 4-5, 5-10, 10-20 or 20-30 d before sample collection | W | - | [newa.cornell.edu](http://newa.cornell.edu/) |  |

^a^ To assess the relative information gain associated with using different data types to build predictive models, two sets of analyses were performed. In the first set of analyses, each learner and the full set of factors listed in Table S1 were used to develop the full models. In the second set of analyses, the factors listed in Table S1 were divided into four groups: geospatial (Gs); physicochemical water quality, and temperature data collected on-site (Pq); weather data obtained from NEWA weather stations (W); and stream traits (St). Nested models were then built using different combinations of these four data types. Separately, log-linear models were also built using individual physicochemical (conductivity, dissolved oxygen, pH, turbidity, or water temperature) or weather (air temperature at the time of sample collection or rainfall 0-1 d before sample collection) factors, or using air temperature at collection, rainfall 0-1d before sample collection, and turbidity.

^b^ New York State = NYS

^c^ For each land cover class we calculated the proportion of (i) the total watershed area, (ii) the stream corridor (i.e., area 0-60 m from the stream corridor), (iii) the flood plain (based on shapefile downloaded from NYS Department of Environmental Conservation), and (iv) the area immediately upstream (0-100 m) of the sampling site.

Table S2: Performance measures for each model. The 10 best and 10 worst performing models (based on root mean squared-error ranking) are denoted in red and goldenrod, respectively.

|  | **Algorithm** | **RMSE ^a^** | **Kendall's Tau** | **R^2^** | **Model (Full Model, or**  **Predictors used to Build Nested Models)** | **Model Rank Based on RMSE (Best Model=117)** |
| --- | --- | --- | --- | --- | --- | --- |
| Baseline | |  |  |  |  |  |
|  | Featureless (Baseline) | 0.51 | NA | -0.02 | Full Model | 97 |
| Forest and Ensemble Algorithms | | | | | |  |
|  | Node Harvest | 0.44 | 0.44 | 0.26 | Full Model | 102 |
|  | Conditional Forest (condRF) | 0.38 | 0.55 | 0.44 | Full Model | 113 |
|  | Extreme Gradient Boosting (XgBoost) | 0.37 | 0.53 | 0.45 | Full Model | 116 |
|  | Random Forest (RF) | | | | |  |
|  |  | 0.38 | 0.54 | 0.45 | Full Model | 114 |
|  |  | 0.83 | 0.29 | 0.14 | Geospatial | 40 |
|  |  | 0.76 | 0.44 | 0.29 | Physicochemical & Geospatial | 67 |
|  |  | 0.77 | 0.42 | 0.27 | Physicochemical | 61 |
|  |  | 0.78 | 0.4 | 0.26 | Physicochemical & Stream Traits | 59 |
|  |  | 0.71 | 0.46 | 0.37 | Physicochemical & Weather | 87 |
|  |  | 0.71 | 0.47 | 0.38 | Physicochemical Water, Weather & Stream Traits | 89 |
|  |  | 0.90 | 0.23 | 0.00 | Stream Traits | 17 |
|  |  | 0.81 | 0.29 | 0.19 | Weather | 44 |
|  |  | 0.74 | 0.48 | 0.33 | Weather & Geospatial | 80 |
|  |  | 0.84 | 0.27 | 0.14 | Weather & Stream Traits | 39 |
|  | Regularized Random Forest (regRF) | | | | |  |
|  |  | 0.38 | 0.54 | 0.45 | Full Model | 115 |
|  |  | 0.84 | 0.29 | 0.12 | Geospatial | 35 |
|  |  | 0.74 | 0.47 | 0.32 | Physicochemical & Geospatial | 77 |
|  |  | 0.78 | 0.41 | 0.25 | Physicochemical | 57 |
|  |  | 0.78 | 0.40 | 0.25 | Physicochemical & Stream Traits | 58 |
|  |  | 0.72 | 0.46 | 0.37 | Physicochemical & Weather | 85 |
|  |  | 0.71 | 0.46 | 0.37 | Physicochemical Water, Weather & Stream Traits | 88 |
|  |  | 0.91 | 0.23 | -0.02 | Stream Traits | 15 |
|  |  | 0.81 | 0.29 | 0.19 | Weather | 45 |
|  |  | 0.76 | 0.42 | 0.29 | Weather & Geospatial | 68 |
|  |  | 0.84 | 0.27 | 0.13 | Weather & Stream Traits | 36 |
| Instance-based Algorithms | | | | |  |  |
|  | K-Nearest Neighbor (kKNN) | 0.90 | 0.27 | 0.00 | Full Model | 18 |
|  | Weighted k-Nearest Neighbor (wKNN) | | | | |  |
|  |  | 0.86 | 0.3 | 0.09 | Full Model | 28 |
|  |  | 0.86 | 0.29 | 0.09 | Geospatial | 30 |
|  |  | 0.86 | 0.31 | 0.09 | Physicochemical & Geospatial | 29 |
|  |  | 0.82 | 0.4 | 0.17 | Physicochemical | 42 |
|  |  | 0.85 | 0.30 | 0.10 | Physicochemical & Stream Traits | 33 |
|  |  | 0.82 | 0.30 | 0.16 | Physicochemical & Weather | 41 |
|  |  | 0.87 | 0.27 | 0.08 | Physicochemical Water, Weather & Stream Traits | 26 |
|  |  | 0.96 | 0.19 | -0.13 | Stream Traits | 7 |
|  |  | 0.93 | 0.07 | -0.06 | Weather | 12 |
|  |  | 0.87 | 0.26 | 0.07 | Weather & Geospatial | 25 |
|  |  | 0.96 | 0.09 | -0.14 | Weather & Stream Traits | 6 |
| Neural Network (NNET) | | 0.75 | 0.45 | 0.30 | Full Model | 74 |
| Penalized Regression | | | | | |  |
|  | Elastic Net | | | | |  |
|  |  | 0.42 | 0.45 | 0.32 | Full Model | 107 |
|  |  | 0.86 | 0.26 | 0.09 | Geospatial | 27 |
|  |  | 0.77 | 0.41 | 0.26 | Physicochemical & Geospatial | 60 |
|  |  | 0.73 | 0.43 | 0.34 | Physicochemical | 81 |
|  |  | 0.74 | 0.43 | 0.33 | Physicochemical & Stream Traits | 79 |
|  |  | 0.71 | 0.48 | 0.38 | Physicochemical & Weather | 90 |
|  |  | 0.72 | 0.47 | 0.36 | Physicochemical Water, Weather & Stream Traits | 84 |
|  |  | 0.91 | 0.23 | -0.03 | Stream Traits | 14 |
|  |  | 0.75 | 0.42 | 0.31 | Weather | 76 |
|  |  | 0.76 | 0.43 | 0.29 | Weather & Geospatial | 66 |
|  |  | 0.79 | 0.37 | 0.23 | Weather & Stream Traits | 51 |
|  | Lasso | 0.42 | 0.47 | 0.33 | Full Model | 108 |
|  | Ridge | 0.42 | 0.43 | 0.31 | Full Model | 106 |
| Regression | |  |  |  |  |  |
|  | Multivariate Adaptive Regression Splines (MARS) | | | | |  |
|  |  | 0.49 | 0.26 | 0.06 | Full Model | 99 |
|  |  | 0.99 | 0.25 | -0.2 | Geospatial | 4 |
|  |  | 0.8 | 0.47 | 0.21 | Physicochemical & Geospatial | 48 |
|  |  | 0.8 | 0.47 | 0.21 | Physicochemical | 49 |
|  |  | 0.8 | 0.47 | 0.21 | Physicochemical & Stream Traits | 47 |
|  |  | 0.76 | 0.4 | 0.29 | Physicochemical & Weather | 69.5 |
|  |  | 0.76 | 0.4 | 0.29 | Physicochemical Water, Weather & Stream Traits | 69.5 |
|  |  | 0.93 | 0.09 | -0.08 | Stream Traits | 9.5 |
|  |  | 0.9 | 0.19 | 0.01 | Weather | 19 |
|  |  | 0.88 | 0.26 | 0.06 | Weather & Geospatial | 23 |
|  |  | 0.91 | 0.22 | -0.01 | Weather & Stream Traits | 16 |
|  | Partial Least Squares (PLS) | | | |  |  |
|  |  | 0.52 | 0.37 | -0.04 | Full Model | 94 |
|  |  | 0.86 | 0.23 | 0.09 | Geospatial | 31 |
|  |  | 0.87 | 0.35 | 0.07 | Physicochemical & Geospatial | 24 |
|  |  | 0.73 | 0.44 | 0.34 | Physicochemical | 82 |
|  |  | 0.76 | 0.41 | 0.3 | Physicochemical & Stream Traits | 72 |
|  |  | 0.71 | 0.48 | 0.39 | Physicochemical & Weather | 91 |
|  |  | 0.69 | 0.5 | 0.41 | Physicochemical Water, Weather & Stream Traits | 93 |
|  |  | 0.95 | 0.23 | -0.11 | Stream Traits | 8 |
|  |  | 0.75 | 0.39 | 0.30 | Weather | 73 |
|  |  | 0.78 | 0.45 | 0.24 | Weather & Geospatial | 56 |
|  |  | 0.8 | 0.34 | 0.21 | Weather & Stream Traits | 50 |
|  | Principal Component (PCR) | 0.51 | 0.36 | -0.02 | Full Model | 95 |
|  | Log-Linear Regression | | | |  |  |
|  |  | 0.89 | 0.16 | 0.02 | Air Temperature at Collection | - |
|  |  | 0.90 | 0.05 | 0.00 | Conductivity | - |
|  |  | 0.86 | 0.36 | 0.08 | Dissolved Oxygen | - |
|  |  | 0.81 | 0.35 | 0.19 | pH | - |
|  |  | 0.90 | 0.14 | 0.00 | Rainfall 0-1 d before Collection | - |
|  |  | 0.78 | 0.45 | 0.25 | Air Temperature at Collection, Rainfall 0-1 d before Collection, & Turbidity | - |
|  |  | 0.74 | 0.44 | 0.32 | Turbidity | - |
|  |  | 0.90 | 0.16 | 0.01 | Water Temp | - |
| Rule-based Algorithms | | | | |  |  |
|  | Cubist (Cubist) | 0.45 | 0.42 | 0.22 | Full Model | 100 |
|  | Cubist, Boosted (BoostedCub) | 0.40 | 0.48 | 0.39 | Full Model | 111 |
|  | Cubist, Boosted k-Nearest Neighbor (Boosted kNCub) | | | | |  |
|  |  | 0.37 | 0.53 | 0.47 | Full Model | 117 |
|  |  | 0.93 | 0.28 | -0.06 | Geospatial | 13 |
|  |  | 0.84 | 0.44 | 0.12 | Physicochemical & Geospatial | 34 |
|  |  | 0.84 | 0.38 | 0.14 | Physicochemical | 38 |
|  |  | 0.84 | 0.41 | 0.13 | Physicochemical & Stream Traits | 37 |
|  |  | 0.82 | 0.36 | 0.18 | Physicochemical & Weather | 43 |
|  |  | 0.76 | 0.41 | 0.3 | Physicochemical Water, Weather & Stream Traits | 71 |
|  |  | 0.99 | 0.18 | -0.21 | Stream Traits | 3 |
|  |  | 0.89 | 0.24 | 0.02 | Weather | 20 |
|  |  | 0.71 | 0.48 | 0.37 | Weather & Geospatial | 86 |
|  |  | 1.00 | 0.16 | -0.22 | Weather & Stream Traits | 2 |
|  | Cubist, k-Nearest Neighbor (kNCub) | 0.42 | 0.46 | 0.31 | Full Model | 105 |
| Support Vector Machines (SVM) | | | | |  |  |
|  | Linear Kernel |  |  |  |  |  |
|  |  | 0.41 | 0.46 | 0.34 | Full Model | 109 |
|  |  | 0.86 | 0.25 | 0.10 | Geospatial | 32 |
|  |  | 0.77 | 0.40 | 0.28 | Physicochemical & Geospatial | 64 |
|  |  | 0.73 | 0.45 | 0.35 | Physicochemical | 83 |
|  |  | 0.76 | 0.38 | 0.28 | Physicochemical & Stream Traits | 65 |
|  |  | 0.70 | 0.49 | 0.40 | Physicochemical & Weather | 92 |
|  |  | 0.74 | 0.43 | 0.32 | Physicochemical Water, Weather & Stream Traits | 78 |
|  |  | 0.93 | 0.23 | -0.06 | Stream Traits | 11 |
|  |  | 0.75 | 0.42 | 0.31 | Weather | 75 |
|  |  | 0.78 | 0.43 | 0.24 | Weather & Geospatial | 55 |
|  |  | 0.81 | 0.32 | 0.20 | Weather & Stream Traits | 46 |
|  | Polynomial Kernel | 0.45 | 0.41 | 0.23 | Full Model | 101 |
|  | Radial Kernel | 0.40 | 0.47 | 0.38 | Full Model | 110 |
|  | Sigmoid Kernel | 0.43 | 0.44 | 0.28 | Full Model | 104 |
| Tree-based Algorithms | | | | |  |  |
|  | Conditional Inference Tree (cTree) | | | | |  |
|  |  | 0.43 | 0.48 | 0.28 | Full Model | 103 |
|  |  | 0.96 | 0.09 | -0.14 | Geospatial | 5 |
|  |  | 0.78 | 0.46 | 0.24 | Physicochemical & Geospatial | 54 |
|  |  | 0.79 | 0.46 | 0.24 | Physicochemical | 52.5 |
|  |  | 0.79 | 0.46 | 0.24 | Physicochemical & Stream Traits | 52.5 |
|  |  | 0.77 | 0.48 | 0.28 | Physicochemical & Weather | 62.5 |
|  |  | 0.77 | 0.48 | 0.28 | Physicochemical Water, Weather & Stream Traits | 62.5 |
|  |  | 0.93 | 0.09 | -0.08 | Stream Traits | 9.5 |
|  |  | 0.88 | 0.22 | 0.04 | Weather | 21 |
|  |  | 1.03 | 0.12 | -0.31 | Weather & Geospatial | 1 |
|  |  | 0.88 | 0.22 | 0.05 | Weather & Stream Traits | 22 |
|  | Evolutionary Optimal Trees (EvTree) | 0.49 | 0.22 | 0.05 | Full Model | 98 |
|  | Extremely Randomized Trees (exTree) | 0.39 | 0.51 | 0.42 | Full Model | 112 |
|  | Regression Tree (CART) | 0.51 | 0.15 | -0.02 | Full Model | 96 |

^a^ Root-mean squared error

**References for Supplemental Materials:**

Bridge Inventory Manual (2006). Available at: https://www.dot.ny.gov/divisions/engineering/structures/repository/manuals/inventory/2006_nysdot_inventory_manual_r.pdf [Accessed June 4, 2019].

Culvert inventory and inspection manual (2006). Available at: https://www.dot.ny.gov/divisions/operating/oom/transportation-maintenance/repository/CulvertInventoryInspectionManual.pdf [Accessed June 4, 2019].

Dams Available at: https://gis.ny.gov/gisdata/inventories/details.cfm?DSID=1130 [Accessed June 17, 2019].

Guidance on outfall mapping: What is an outfall, and what should be mapped? (2006). Albany, NY Available at: http://www.dec.state.ny.us/website/dow/MS4crit.pdf. [Accessed June 4, 2019].

Methodology for the identification and survey of stormwater outfalls within designated MS4 locations for New York State DOT (2004). Albany, NY Available at: https://www.dot.ny.gov/divisions/engineering/environmental-analysis/repository/OutfallMethodology.pdf [Accessed June 4, 2019].

National Hydrography Database Available at: https://www.usgs.gov/core-science-systems/ngp/national-hydrography [Accessed June 17, 2019].

NLCD 2016 Land Cover (CONUS) (2019). Sioux Falls, SD, SD Available at: https://www.mrlc.gov/data?f%5B0%5D=category%3Aland cover.

NLCD 2016 Percent Developed Imperviousness (2019). Sioux Falls, SD Available at: https://www.mrlc.gov/data?f%5B0%5D=category%3Aurban imperviousness.

NYS Large Culverts Available at: https://gis.ny.gov/gisdata/inventories/details.cfm?DSID=1255 [Accessed June 17, 2019].

Outfall and system mapping for illicit discharge detection and elimination (IDDE) in NY Albany, NY, NY Available at: ftp://ftp.dec.state.ny.us/dow/stormdocuments/ms4/illicit_discharge_detection_and_elimination/illicit_discharge_detection_and_elimination_assistance/guidance/IDDE NYS Mapping Doc FINAL 06 12 05 for FTP site.pdf.

Septic Systems, New York State, 2011 - CUGIR Available at: https://cugir.library.cornell.edu/catalog/cugir-008164 [Accessed July 29, 2019].

Solid Waste Management Facilities | Open Data NY Available at: https://data.ny.gov/Energy-Environment/Solid-Waste-Management-Facilities/2fni-raj8/data [Accessed June 17, 2019].

State Pollutant Discharge Elimination System Available at: https://gis.ny.gov/gisdata/inventories/details.cfm?DSID=1010 [Accessed June 17, 2019].

Unified Stream Assessment: A User’s Manual (2004). Ellicott City, MD.

USGS National Transportation Dataset Available at: https://catalog.data.gov/dataset/usgs-national-transportation-dataset-ntd-downloadable-data-collectionde7d2 [Accessed June 17, 2019].

Weller, D. L., Love, T. M. T., Belias, A., and Wiedmann, M. (2020). Predictive models may complement or provide an alternative to existing strategies for managing enteric pathogen contamination of Northeastern streams used for produce production. *Front. Sustain. Food Syst.* 4, 561517. doi:10.3389/fsufs.2020.561517.
